# In Silico Evaluation of Nonsynonymous SNPs in Human ADAM33: The Most Common Form of Genetic Association to Asthma Susceptibility

**DOI:** 10.1155/2022/1089722

**Published:** 2022-11-12

**Authors:** Milad Mohkam, Nasim Golkar, Seyed Hesamodin Nabavizadeh, Hossein Esmaeilzadeh, Aydin Berenjian, Zahra Ghahramani, Ahmad Gholami, Soheila Alyasin

**Affiliations:** ^1^Allergy Research Center, Shiraz University of Medical Sciences, Shiraz, Iran; ^2^Pharmaceutical Sciences Research Center, Shiraz University of Medical Sciences, Shiraz, Iran; ^3^Department of Pharmaceutics, School of Pharmacy, Shiraz University of Medical Sciences, Shiraz, Iran; ^4^Department of Allergy and Clinical Immunology, Namazi Hospital, Shiraz University of Medical Sciences, Shiraz, Iran; ^5^School of Engineering, University of Waikato, Hamilton 3240, New Zealand; ^6^School of Medicine, Shiraz University of Medical Sciences, Shiraz, Iran; ^7^Biotechnology Research Center, Shiraz University of Medical Sciences, Shiraz, Iran

## Abstract

ADAM33 is a zinc-dependent metalloprotease of the ADAM family, which plays a vital biological role as an activator of Th_2_ cytokines and growth factors. Moreover, this protein is crucial for the normal development of the lung in the fetus two months after gestation leading to determining lung functions all over life. In this regard, mutations in ADAM33 have been linked with asthma risk factors. Consequently, identifying ADAM33 pathogenic nonsynonymous single-nucleotide polymorphisms (nsSNPs) can be very important in asthma treatment. In the present study, 1055 nsSNPs of human ADAM33 were analyzed using biocomputational software, 31 of which were found to be detrimental mutations. Precise structural and stability analysis revealed D219V, C669G, and C606S as the most destabilizing SNPs. Furthermore, MD simulations disclosed higher overall fluctuation and alteration in intramolecular interactions compared with the wild-type structure. Overall, the results suggest D219V, C669G, and C606S detrimental mutations as a starting point for further case-control studies on the ADAM33 protein as well as an essential source for future targeted mechanisms.

## 1. Introduction

Asthma is a multifaceted lifetime pulmonary disease of the respiratory bronchi, described by fluctuating and repetitive symptoms, bronchi spasms, and reversible airflow obstruction [1]. It consists of inflammation and swelling of the bronchi leading to perpetual scarring/remodeling of the bronchi. The indications of asthma comprise occurrences of wheezing, coughing, chest tightness, and breathlessness, which are a result of alterations in the constitutes of cellular and extracellular matrix in the small and large airways, apoptosis of epithelial cells, activation of fibroblast, and proliferation of airway smooth muscle cells [2, 3]. Contingent upon the patients, the indications of asthma may become severe by exercise or at night time, exerting a remarkable burden on life quality, work efficiency, social activity, and healthcare resource use [4, 5].

Consequently, there has been an increase in prevalence rates, albeit with a decline in asthma mortality ratios in several countries in recent years. For instance, 345 million people worldwide had asthma in 2019, compared to 183 million patients in 1990 [6]. Although numerous therapeutics are now extensively utilized owing to their superior efficiency and fewer side effects in asthma treatment, all of these drugs are accessible in a prescription manner [7, 8]. Moreover, a notably high incidence of scantily controlled asthma has been recognized. For a number of asthmatic patients (5–10%), the illness is obstinate to corticosteroid healing and regularly causes hospitalization, thanks to rhinovirus respiratory infection. Furthermore, asthma indications initiated in adults could have resulted from childhood [9]; however, if bronchial function no longer reoccurs into the normal status when there is no attack, the asthma is eventually categorized as a chronic obstructive pulmonary disease (COPD). Thus, there is a high-priority requirement to recognize various significant biomarkers that can aid in predicting this disorder and guide curative policies.

Many genetic investigations have been carried out to recognize genetic polymorphisms related to asthma vulnerability. In this regard, a gene called metalloprotease and disintegrin 33 (ADAM33), placed on human chromosome 20p13, is among the initially recognized asthma nominee genes. It belongs to the zinc-dependent metalloproteases of the ADAM family and participates in a vital biological task as an activator of Th_2_ cytokines and growth factors [10]. ADAM33 possesses 22 exons for encoding a catalytic domain, predomain, signal sequence, cysteine-rich domain, disintegrin domain, transmembrane domain, cytoplasmic domain, and EGF domain with a 3′-untranslated region (UTR) [11]. The mentioned diverse domains provide distinctive ADAM33 biological functions involving proteolysis, cell activation and fusion, intracellular signaling, and adhesion [12].

Furthermore, genetic investigations have revealed that ADAM33 can take part in ascertaining lung function during life, correlating with the elevated prospect of curative intervention in asthma [13]. Moreover, it is reported that soluble ADAM33 protein can enhance angiogenesis, which could be regarded as a “tissue remodeling gene” by affecting lung functions and obstruction of airflow separately from inflammation [14]. Evidence has demonstrated that ADAM33 can be a vulnerable target gene in asthma [15] and have a vital function in the natural history as well as the asthma origins [16]. In addition, the ADAM33 mRNA that expresses preferentially in myofibroblasts, fibroblasts, and smooth muscles indicates that its abnormality functions would be possibly related to airway wall “remodeling” and bronchial hyperresponsiveness (BHR), which leads to the asthma disorder in the early life of individuals. Furthermore, a superior ADAM33 protein expression was found in asthmatic patients in comparison to the control groups [17].

In compliance with various investigations, single-nucleotide polymorphisms (SNPs) in the ADAM33 gene can hinder the physiological functions deployed through the ADAM33 protein. A current extensive meta-analysis of populations has indicated the relationship between genetic variation with asthma progression [18]. In this context, F +1, T2, and Q1 polymorphisms of the ADAM33 gene may help to cause asthma risk in Asian populations, while V4 polymorphism usually occurs in Caucasian populations [18, 19]. Although several reports outline the interrelation of the ADAM33 gene with asthma disease, no in-depth research has been conducted either computationally or experimentally to inspect the importance of these polymorphisms' structural and functional states. Due to highly costly and time-consuming *in vivo* studies, computational approaches to functional nonsynonymous single-nucleotide polymorphisms (nsSNPs) have been regarded as an intelligent, helpful procedure before conducting empirical research [20]. In this regard, the in silico evaluation of ADAM33 as one of the chief causative parameters of asthma was undertaken to investigate the potential risk of ADAM33 polymorphisms from the vast number of neutral SNPs of the ADAM33 gene in the induction of asthma disorder according to the available databases as well as to attain a more dependable outcome.

## 2. Materials and Methods

**Figure 1:**
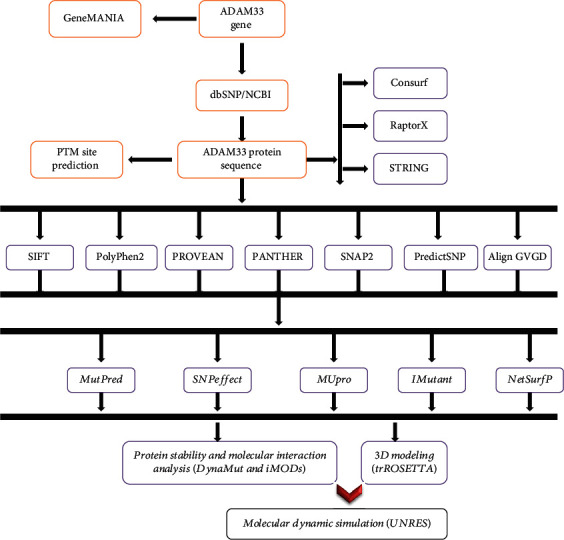
Schematic representation of the whole analytic procedure of the study.

The entire approach utilized in the present research is briefly illustrated in a flowchart (Figure 1).

### 2.1. ADAM33 Protein Characterization and SNP Data Retrieval

The protein sequence information of the ADAM33 gene (ID: Q9BZ11) was attained from the UniProtKB database with the address http://www.uniprot.org/uniprot/. In addition, the information relating to ADAM33 protein was obtained from InterPro databases (database of protein families, functional sites, and protein domains) (https://www.ebi.ac.uk/interpro/) [21]. Moreover, the prediction for the property of the ADAM33 structure was carried out by RaptorX property (http://raptorx.uchicago.edu/StructurePropertyPred/predict/) [22].

The SNP data of the human ADAM33 gene was attained from dbSNP-NCBI with the address http://www.ncbi.nlm.nih.gov/SNP/, and the nsSNPs were filtered out for additional studies.

### 2.2. Prediction of Deleteriousness of SNPs

The structural and functional impacts of damaging SNPs of the ADAM33 gene were analyzed using SIFT, PolyPhen-2, PROVEAN, PANTHER, SNAP2, Align GVGD, and PredictSNP online web tools, sequentially.

The functional influences of nsSNP were predicted by applying various computational tools. Prediction of the harmful impacts of nsSNPs (http://sift.jcvi.org/) was carried out via the Sorting Intolerant from Tolerant (SIFT) online web server. SIFT employs the sequence homology-based method to predict an amino acid substitution that impacts protein function via calculating the amino acid conservation degree throughout evolution [23]. The SIFT scores ranged from 0 to 1, in which the value below 0.05 implied the destructive impact of nsSNPs on protein function or structure. Another prediction tool exploited to investigate the functional influences of nsSNPs was PolyPhen-2 with the address http://genetics.bwh.harvard.edu/pph2, which utilizes a variety of structure- and sequence-based comparisons for prediction of the consequences of nsSNP on both protein function and structure [24]. The prediction results were attained in the type of probability scores categorized into three categories: “benign,” “possibly damaging,” and “probably damaging,” and the cutoff value was set for “probably damaging,” with a score above 0.95.

An online server of PROVEAN with the address http://provean.jcvi.org/seq_submit.php is based on a sequence homology approach (using a delta alignment score) for the prediction of the functional consequence of an amino acid substitution (including substitutions, deletions, and insertions) [25]. The cutoff value adjusted for the PROVEAN server is -6.

The PANTHER PSEP (protein analysis through evolutionary relationship-coding SNP) web server with the address http://pantherdb.org/tools/csnpScoreForm.jsp determines the effect of nsSNPs on protein function via evolutionary conservation. In addition, this server calculates the alignment of various evolutionarily related proteins to provide the scoring system based on position-specific evolutionary conservation (PSEC) scores provided [26].

The SNAP2 web server (Screening for Nonacceptable Polymorphisms) with the address https://www.rostlab.org/services/SNAP/ discriminates neutral and effect variants via inspecting a range of variants as well as sequence traits, including secondary structure, evolutionary, annotation data, and solvent accessibility [27]. The protein sequences in the FASTA format were incorporated as an input query.

The Align GVGD web server with the address http://agvgd.hci.utah.edu/ is the basis of biophysical traits of amino acids and protein multiple sequence alignments for predicting damaging amino acid substitution in proteins. It provides a variety of sorted variants (C0, C15, C25, C35, C45, C55, and C65) in which C65 is mainly probable to hamper function and vice versa [28].

The PredictSNP online tool (https://loschmidt.chemi.muni.cz/predictsnp1/) gathers the input data from various tools for estimating the consequence of single amino acid substitution. This tool offers a consensus prediction with enhanced efficiency and precision rather than an individual integrated tool [29].

We established a criterion to narrow the obtained predictive outcomes of the online tools. Due to the huge nsSNP input (about 5000 nsSNPs), only the most likely deleterious or damaging variations (the highest scores) predicted by all the used web servers were selected for subsequent analysis like structure stability changes, energy changes, and surface accessibility using different tools.

### 2.3. Prediction of Stability Alteration of the Mutant Protein

The stability of the mutant protein was verified utilizing five servers: IMutant2.0, MutPred2, MUpro, NetSurfP.2, and SNPeffect.I-Mutant2.0 with the address http://folding.biofold.org/i-mutant/i-mutant2.0.html [30]. The consequence of amino acid substitution (AAS) for the prediction of the functional and structural alterations was assessed by the MutPred2 server with the address http://mutpred.mutdb.org/ [31]. MUpro (http://mupro.proteomics.ics.uci.edu/) is an online software tool used to predict stability changes of single point mutation based on a method named support vector machine (SVM) [32]. In order to predict the secondary structure as well as the surface accessibility of amino acids, the NetSurfP server (http://www.cbs.dtu.dk/services/NetSurfP/), which is a neural network-based algorithm, was used accordingly [33]. The SNPeffect 4.0 server with the address http://snpeffect.switchlab.org/ offers structure- and sequence-based methods to predict the impact of nsSNPs on the structure and function of human proteins [34]. SNP effect utilized four tools, including TANGO (aggregation prediction), WALTZ (amyloid prediction), LIMBO (chaperone-binding prediction), and FoldX (protein stability analysis) for protein structure phenotyping [34].

### 2.4. Prediction of Ligand-Binding Sites

Ligand-binding sites of ADAM33 protein were forecasted using the online server RaptorX binding with the address http://raptorx.uchicago.edu/BindingSite/. This online tool calculates the pocket multiplicity along with *P* value, uSeqID (SeqID), and uGDT (GDT), which is utilized to conclude the predicted pocket quality. In this context, the superior score is implied for the more precise predicted pocket, particularly when the score reaches over 40 [35].

### 2.5. Analysis of Gene-Gene Interaction

The gene-gene interactions were performed to highlight nominee genes that might be related to asthma disease. The GeneMANIA online tool (http://genemania.org/) can discover other genes related to a group of input ones via a massive series of functional association information. Association data comprises genetic and protein interactions, protein domain similarity, coexpression, pathways, and colocalization [36].

### 2.6. Analysis of Protein-Protein Interaction

The STRING server with the address https://string-db.org/cgi/input?sessionId=bEbFMsdUTTLq&input_page_show_search=on was employed to analyze the interaction between proteins. This database provides an imperative evaluation and integration of protein-protein interaction for easy access to validated theoretical and experimental interactions of the desired protein [37].

### 2.7. 3D Protein Modeling and Quality Evaluation of the Modeled Proteins

Wild types and mutants recognized by a superior impact upon point mutation through an upstream analysis were modeled using trROSETTA. This modeling software predicts structures from sequence information using a deep learning tool via ab initio folding [38]. The obtained top models were subjected to the 3Drefine server with the address of http://sysbio.rnet.missouri.edu/3Drefine/ for structure refinement [39].

The quality of the obtained protein models was further assessed by ProSA-web, (https://prosa.services.came.sbg.ac.at/prosa.php) [40], PROCHECK (https://servicesn.mbi.ucla.edu/PROCHECK/) [41], ERRAT (http://servicesn.mbi.ucla.edu/ERRAT/) [42], and Verify3D (http://servicesn.mbi.ucla.edu/Verify3D/) [43]. Finally, the TM-align tool assessed the mutated models with the address https://zhanglab.dcmb.med.umich.edu/TM-align/ to evaluate the structural deviation degree among natives and mutants [44]. In this regard, template modeling scores named TM score and root mean square deviation named RMSD values were calculated to compare the protein structures on the basis of the structural superimpositions to discover the structure's similarity. TM scores are from 0 to 1, in which 1 indicates a complete match between two structures. A TM score between 0.0 and 0.30 signifies random structural similarity, while a TM score between 0.5 and 1.00 signifies that both structures are in the equal fold [44].

### 2.8. Prediction of Alterations in Protein Stability and Interaction upon nsSNPs

Prediction of alterations in the stability of protein and interaction upon nsSNPs were carried out using the DynaMut server with the address http://biosig.unimelb.edu.au/dynamut/. This server performed prediction by evaluating flexibility analysis and protein dynamics [45].

### 2.9. Normal Mode Analysis

Normal mode analysis was carried out through the iMOD server (iMODs) (http://imods.chaconlab.org), in which the preset values of variables were utilized. This online tool is a rapid and user-friendly molecular dynamic simulation software program that can swiftly characterize potential conformational alterations [46].

### 2.10. Molecular Dynamic (MD) Simulation

Molecular dynamic simulation of the best models attained by validation web tools was subjected to the dynamic stability analysis of the wild and mutated proteins using the UNRES online server with the address http://unres-server.chem.ug.edu.pl/ [47, 48]. This server predicts the thermodynamics and dynamic of proteins on the basis of physics-based coarse-grained simulations. Moreover, it can be applied to forecast dynamics, interactions, and protein structure with superb precision at larger times [49–51]. The coarse-grain-based MD approach was run using the default values of parameters. MD runs were executed to predict fluctuation, potential energy, and the radius of gyration.

### 2.11. Ethical Approval

All authors declared that no human or animal study was included throughout this study. This study was approved by the bioinformatic grant committee of Shiraz University of Medical Sciences, Shiraz, Iran.

## 3. Results

### 3.1. ADAM33 Protein Characterization and SNP Data Retrieval

The human ADAM33 gene contains 23,575 kbp, and its protein consists of 813 amino acids. The existence of functional domains in ADAM33 protein including Peptidase_M12b_N (56-152), Reprolysin (210-409), Disintegrin_dom (417-503), ADAM-Cys_rich (502-645), EGF-like_dom (649-681), signal peptide (1-27), and cytoplasmic domains (726-813) was investigated in InterPro and UniProtKB databases. The structural information obtained from the UniProtKB database demonstrated distinctive parts involving an extracellular domain (30-701), a transmembrane domain with helical structure (702-722), and a cytoplasmic domain (723-813).

The RaptorX server predicted 15% *α*-helix, 17% *β*-sheet, and 67% coil for the ADAM33 protein. Moreover, there were three states of residue-relevant solvent accessibility, i.e., exposed, medium, and buried, which comprised 51%, 28%, and 20% of the ADAM33 protein, respectively. Moreover, a total of 188 residues (22%) were predicted as disordered.

**Figure 2:**
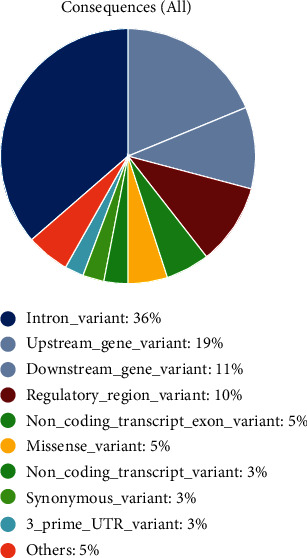
Distribution of various types of SNPs recognized in the ADAM33 gene.

A total of 7522 SNPs for the ADAM33 protein were achieved from the dbSNP database in which nonredundant SNPs were only considered. The recognized SNPs were categorized into various functional classes involving inframe deletion [13], inframe indel [9], inframe insertion [13], initiator codon variant [7], intron (4791), missense (1055), noncoding transcript variant (1772), and synonymous (558) SNPs. Most SNPs belonged to intronic SNP, followed by the noncoding transcript variant and missense SNPs. The distribution of SNPs is illustrated in Figure 2. In this research, only nonsynonymous SNPs were regarded for additional investigations.

### 3.2. Prediction of Deleterious SNPs

Seven tools, including SIFT, PolyPhen-2, PROVEAN, PANTHER, SNAP2, Align GVGD, and PredictSNP, were recruited for pathogenicity or deleterious prediction of nsSNPs by setting limitation criteria for all of the abovementioned tools due to the large number of nsSNPs. Accordingly, 31 nsSNPs among 1055 were forecasted unanimously to be deleterious nsSNPs in all bioinformatic tools (Table 1).

### 3.3. Prediction of Stability Alteration of the Mutant Protein

For forecasting the alterations in the stability of ADAM33 protein, the 31 mutants were selected from the previous step and subjected to structural evaluation using five distinctive web server tools. The obtained outcomes are tabulated in Table 2.

### 3.4. SNPeffect 4.0

The outcome of SNPeffect 4.0 was obtained from 4 different structure- and sequence-based bioinformatic tools comprising TANGO, WALTZ, LIMBO, and FoldX. TANGO predicted the tendency for protein aggregation (Table 3). In this study, only one mutation (D219V) was classified to increase protein aggregation, and the rest did not alter the protein aggregation from the wild-type one. WALTZ predicted the amyloid propensity of protein, which is more accurate and specific than the TANGO algorithm. Only two mutations (C444Y and C388Y) were responsible for rising amyloid tendency, while one mutation (D219V) resulted in its decrease. On the other hand, no mutation was classified as inducing alterations in chaperone binding compared to the wild-type ADAM33. FoldX estimates the discrepancy in each mutation's free energy (ddG). Any rise in the ddG value implies destabilization of the ADAM33 protein and vice versa upon mutation. In this regard, only five mutations were accountable for reducing protein stability. The rest of the mutations did not lead to protein stability change due to a lack of reliable structural information.

### 3.5. MUpro2 and IMutant2.0

MUpro and IMutant2.0 predicted any stability change in the ADAM33 protein, where all nsSNPs decreased the stability of the ADAM33 protein. In contrast, only P678L and C573Y SNPs were predicted to increase stability, as identified by MUpro2 and IMutant2.0 software programs (Table 2).

### 3.6. MutPred2

The results of selected nsSNPs by MutPred software demonstrated the probability of damaging the protein and possibly altering protein function. It was found that all mutations were damaging for ADAM33 protein with a score of more than 0.5 (0.62-0.93) as well as *P* value below 0.05. In this regard, the scores with *P* < 0.05 and *g* > 0.5 designate an actionable hypothesis, the scores with *P* < 0.05 and *g* > 0.75 designate a confident hypothesis, and the scores with *P* < 0.01 and *g* > 0.75 designate a very confident hypothesis due to the nonsynonymous mutation on the basis of the mechanistic disruption. Therefore, all selected nsSNPs were regarded as a very confident hypothesis except for only the two mutations of C637Y and C371Y, which were considered a confident hypothesis. In addition, various molecular mechanism alterations were discovered, including altered transmembrane protein, altered metal binding, loss of catalytic site, and loss of disulfide linkage (Supplementary Table S1).

### 3.7. NetSurfP

NetSurfP online software was used for the solvent accessibility of the ADAM33 protein. In this regard, the class changes from the exposed state to the buried one and the buried state to the exposed one were provided. The results showed that only the two mutations, including P678L and C360R, took part in class alignment change from the buried state to the exposed state with increasing RSA value, implying an increase in solvent accessibility (Table 4).

### 3.8. Ligand-Binding Site Prediction

The RaptorX server was employed to predict ligand-binding sites of ADAM33 protein, followed by an investigation of any mutation within the recognized ligand-binding sites. The results showed that four distinct domains were predicted in the ADAM33 protein (Table 5). The results were provided as four values: uGDT (GDT), uSeqID (SeqID), *P* value, and multiplicity. The threshold for acceptance of the predicted model(s) for each value is as follows: uGDT (GDT) ≥ 50, uSeqID (SeqID) ≥ 30%, *P* value ≤ 10^−3^, and multiplicity ≥ 40. Accordingly, only domain 1 met all the abovementioned criteria and was predicted as a good model. However, domain 4 could be considered a relatively good model despite its lower multiplicity value. Other predicted domains cannot be regarded as correct models due to not passing the criteria mentioned above (except multiplicity). The location of domain 1 is within residues 199-414, which consists of 2 pockets, and the following residues might be prone to mutations: T310, L313, T342, H345, H355, A374, A375, and D296. A similar pattern was also observed for the following residues: L484, E486, D498, V499, L419, N422, E426, E429, D432, and D481. However, no deleterious mutation was observed by our defined restriction criteria.

### 3.9. Prediction of Posttranslational Modification (PTM) Sites

The possible occurrence of posttranslational modification in ADAM33 protein was evaluated using various servers for ubiquitination, glycation, phosphorylation, and sumoylation sites. The BDM-PUB server revealed that 13 residues were predicted to be ubiquitinated (Table S2). However, the upstream analysis reported no mutation in the predicted ubiquitination sites of ADAM33 protein.

The probability of the presence of phosphorylation sites in the protein sequence was evaluated for Thr, Tyr, and Ser residues. The scores above 0.9 were generally regarded as “very probable” to be correct phosphorylation sites. In this regard, 13 out of 93 identified residues were recognized as phosphorylation sites in the ADAM33 protein (Table S3).

GlycoEP predicts N-linked glycosylation, O-linked glycosylation, and C-linked glycosylation for a given protein sequence. This server predicted 5, 13, and 0 sites of N-linked glycosylation, O-linked glycosylation, and C-linked glycosylation, respectively (please refer to Table S4 as a supplementary file).

SUMOs fundamentally regulate different biological processes by adding SUMO-interaction motifs (SIMs) or SUMOylation sites in proteins. Accordingly, no SUMOylation site was predicted by adjusting the threshold to medium and high values by the GPS-SUMO 2.0 online server. However, by adjusting the threshold to the low value, some predicted SUMOylation sites with a *P* value lower than 0.05, implying insignificant or low confidence results (data not shown).

### 3.10. Conservation Analysis

The comprehensive study of evolutionary conservation analysis of 31 nsSNPs using the ConSurf server showed that out of 31 nsSNPs, 17 mutants had a conservation score of 9 (highly conserved residues). The remaining mutants (12 residues) had a conservation score of 8 (relatively highly conserved residues), followed by 2 mutants (conservation score of 7) that were predicted to be moderately conserved. Moreover, ConSurf provided prediction for functional or structural on the basis of solvent accessibility and conservation for amino acid residues. Among these 17 highly conserved residues, 14 mutations were predicted to be structural and buried, and the rest (3 mutations) were predicted to be functional and exposed (Table 6).

### 3.11. Prediction of Gene-Gene and Protein-Protein Interaction

**Figure 3:**
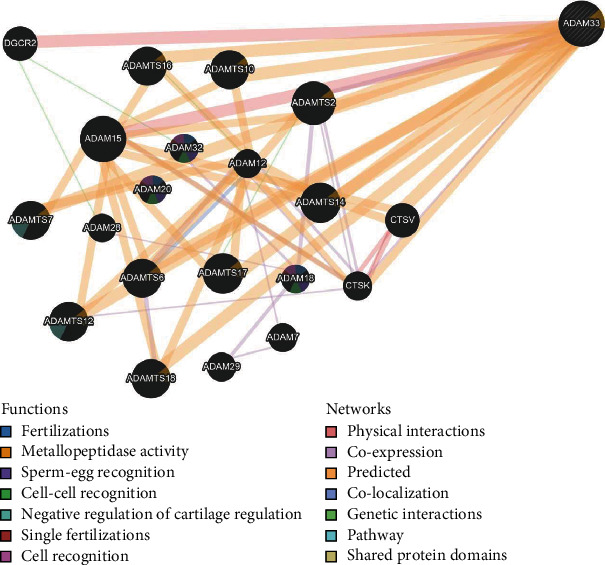
Gene-gene interaction network of ADAM33 protein illustrated by GeneMANIA.

The GeneMANIA tool was used to analyze the gene-gene interaction of the ADAM33 protein. Results showed that the ADAM33 gene has interacted with a number of ADAM families along with DGCR2, CTSK, and CTSV genes. Moreover, the coexpression genes and any contribution to attaining similar functions or sharing similar protein domains are shown in Figure 3.

**Figure 4:**
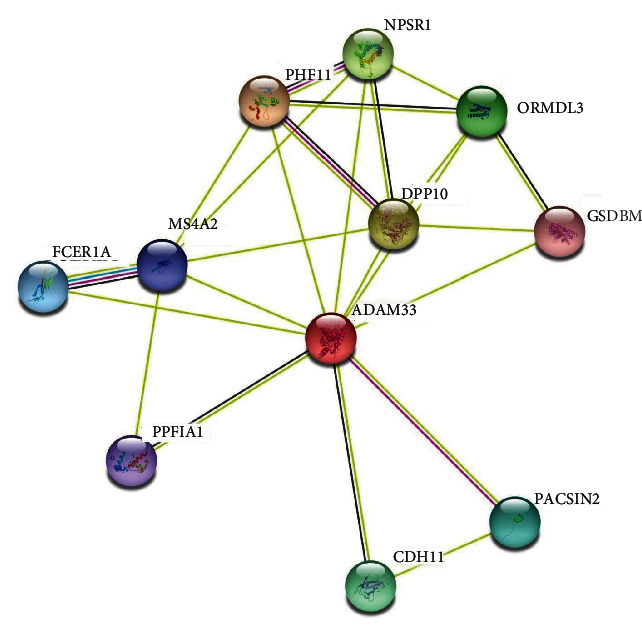
Protein-protein interaction network of ADAM33 using a STRING server.

String revealed that ADAM33 protein has interacted with FCER1*α* (high-affinity immunoglobulin epsilon receptor subunit alpha), MS4A2 (high-affinity immunoglobulin epsilon receptor subunit *β*), GSDMB (Gasdermin-B), and PHF11 (PHD finger protein 11) proteins which take part in immune system functions. Other interactions with proteins are illustrated in Figure 4.

### 3.12. 3D Protein Modeling and Quality Evaluation of Modeled Proteins

In order to determine which of the potential asthma deriver nsSNPs should be subordinated to homology modeling, all evaluation tools were used with a stringent threshold of deleteriousness/effectiveness (elevated dWALTZ, dTANGO, or dLIMBO scores, decreased stability by FoldX evaluation, decreased stability by IMutant and MUpro analysis with RI ≥ 5, a MutPred score of above 0.68, and class alignment changes by NetSurfP analysis) for building a 3D model. In this context, the mutations of D219V, C388Y, C444Y, C475G, C606G, and C669G were analyzed.

**Figure 5:**
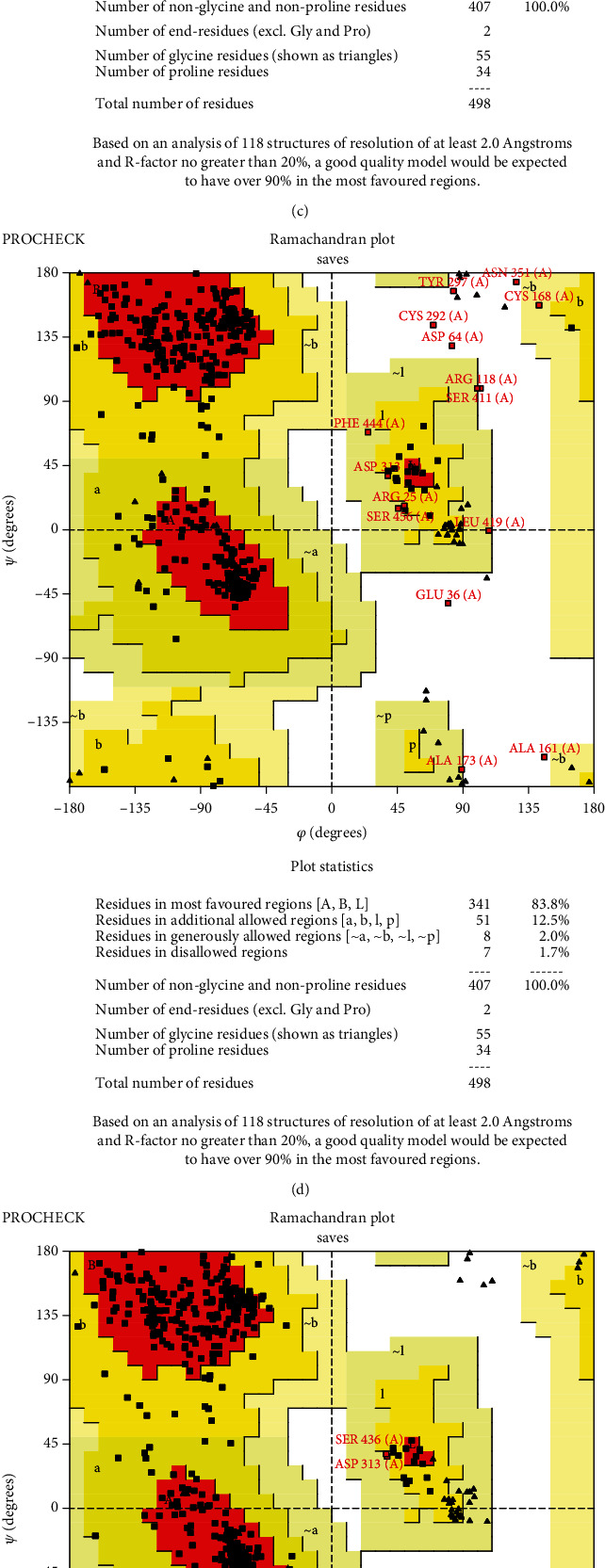
Ramachandran plots of variants generated by PROCHECK. The most favored regions are colored red; additional allowed, generously allowed, and disallowed regions are indicated as yellow, light yellow, and white fields.

Due to the not availability of the 3D structure of full-length ADAM protein in the protein data bank, the FASTA amino acid sequences, as well as mutated protein sequences, were submitted to the trROSETTA server to model the 3D structure of ADAM33 protein. The server provided 5 top models, from which model 1 underwent a structure refinement by the 3Drefine server. The same procedure was done for all mutated proteins. Additional evaluations were performed using PROCHECK, Verify3D, ERRAT, and ProSA programs to calculate the quality of the model, which revealed good results for model 1 (Table 7 and Figure 5). Then, the TM score and RMSD were calculated as standards to determine the structural similarity between the two structures (Table 8). All the predicted mutated models possessed TM scores above 0.5. Moreover, the RMSD measurement was performed to evaluate the variation in the mutant structure compared to the wild-type protein, in which the higher values signify more deviation from the wild-type protein. C669G, followed by C444Y and C475G, had the maximum RMSD values, which signify the significant structural stability of high-risk nsSNPs.

### 3.13. Prediction of Alterations in Stability of Protein and Interaction upon nsSNPs

**Figure 6:**
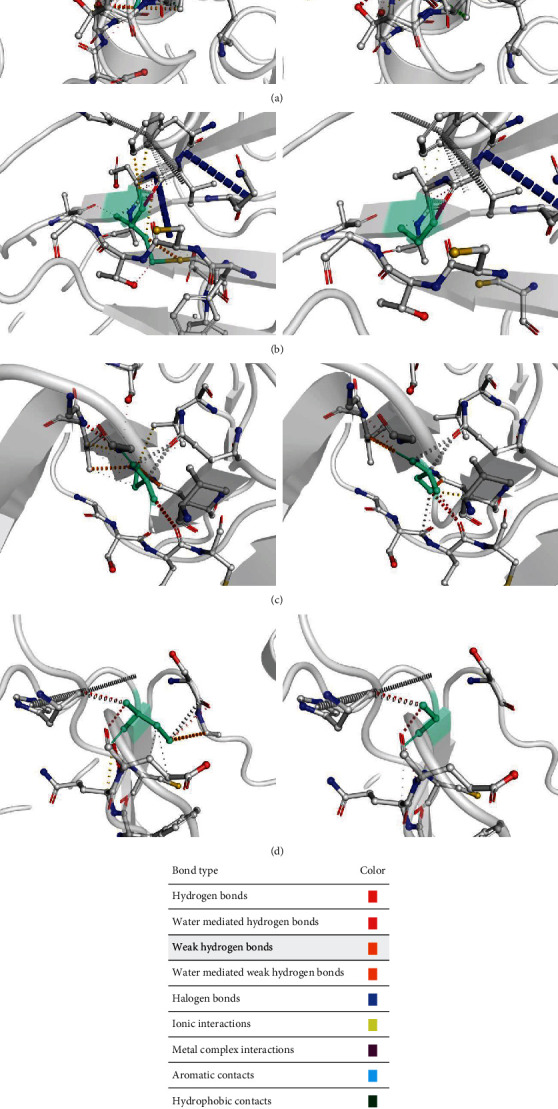
Comparison of interatomic interaction of mutant ADAM33 with the wild-type protein, shown by DynaMut in (a) D219V, (b) C475G, (c) C606S, and (d) C669G. Each section is right and left pictures to demonstrate the mutant and wild-type interactions and represents the color code of interactions.

The DynaMut server was used to calculate general dynamic traits of the highest deleterious nsSNPs selected from the previous analysis steps, including D219V, C444Y, C388Y, C669G, C475G, and C606S mutants. DynaMut portrayed the predictions for Δ entropy energy and ΔΔ*G* by ENCoM among the wild-type and mutant ADAM33 protein. The C669G, C475G, and C606S mutants showed a decrease in the ΔΔ*G* ENCoM value compared to the wild-type SHANK3. On the other hand, the ΔΔ*S* ENCoM value decreased in D219V, C444Y, and C388Y mutants compared to the wild-type protein. Moreover, DynaMut predicted the decrease in ΔΔ*G* for D219V, C669G, C475G, and C606S, implying destabilization (Table 9). Inspection of interatomic interactions was conducted to discover the reasons behind the destabilization of mutant proteins. In this context, the type and the number of interactions changed and decreased, respectively (Figure 6).

### 3.14. Normal Mode Analysis (NMA)

Normal mode analysis was carried out to explain the protein stability and their large-scale mobility. The iMODs tool provided the complete analysis comprising eigenvalues, profiles of mobility (*B*-factors), deformability, covariance map, and linking matrix. The eigenvalue indicates the total mean square fluctuations, which are straightly associated with the energy needed for the deformation of the structure and signify the stiffness of motion. In this regard, the lower eigenvalue implies easier deformation. The outcomes of the iMOD server disclosed that the eigenvalues of D219V, C669G, C388Y, and C606S were lower than wild-type proteins, which points out the distinct behavior of wild-type and mutant proteins (provided as supplementary file in Figures S1-S7).

### 3.15. Molecular Dynamic (MD) Simulation

**Figure 7:**
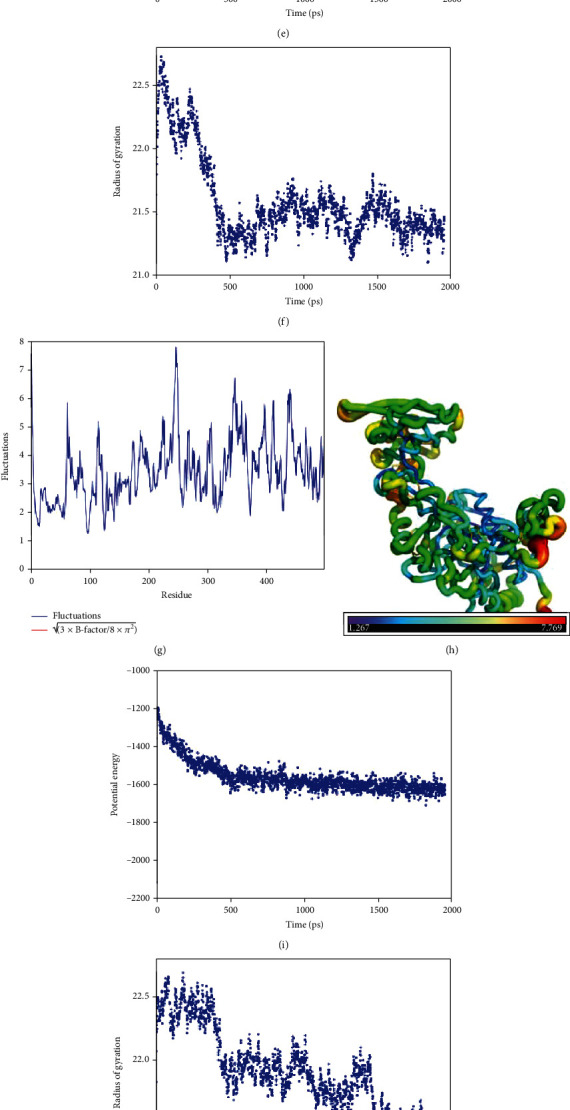
Molecular dynamic simulation analysis of the wild-type and mutant (C669G, C606S, and D219V) variants in a 2000 ps simulation. Plots of potential energy (kcal/mol) for wild type (a), C669G (e), C606S (i), and D219V (m). Plots of the radius of gyration (Å) for wild type (b), C669G (f), C606S (j), and D219V (n). Cartoon putty demonstrations of ADAM33 structures: wild type (c), C669G (g), C606S (k), and D219V (o). Blue signifies the lowest value for *B*-factor, and red is for the highest value. The size of the tube mirrors the value of the *B*-factor (i.e., the thicker the tube is, the greater the *B*-factor). A plot of the residue-based fluctuations (Å): wild type (d), C669G (h), C606S (l), and D219V (p).

The highest detrimental mutations and wild-type protein models were incorporated into the MD simulation. The consensus results of DynaMut and iMOD servers (D219V, C669G, and C606S) were selected for MD simulation. By recruiting coarse-grained (CG) models, additional information was obtained concerning the conformational structure of wild-type ADAM protein and alterations due to the abovementioned mutations in a 2000 ps time frame. Results showed that only the wild-type and C606S substitution demonstrated a small phase of constant decline in the UNRES (united residue) potential energy followed by a palpable steady state, which stayed up to the end of the simulation (Figure 7). However, the rest of the mutations portrayed a steady decline in the UNRES (united residue) potential energy.

On the other hand, the radius of gyration plots which computes protein compactness exhibited that only mutants C669G had an extremely high radius of gyration in comparison to the wild-type protein. Nevertheless, D219V and C606S substitutions had a relatively higher degree of gyration radius than the wild-type protein (Figure 7). Similar patterns were also observed for fluctuation plots for mutated proteins (Figure 7). Moreover, the analysis of atomic fluctuations showed that the overall residue-based flexibility of the mutants' system was elevated compared to the wild-type system (Figure 7). All mutations seemed to modify the intermolecular interactions, which could impair ADAM33 protein function.

## 4. Discussion

Although the ADAM33 protein has been recognized as an asthma vulnerability gene, its function in the progression and pathogenesis of the asthma disorder has not been completely known. ADAM33 is mainly expressed in mesenchymal origin cells, chiefly fibroblasts, myofibroblasts, and smooth muscle cells, signifying a probable function in airway remodeling [52].

In recent years, the existence of detrimental SNPs in a number of asthma-associated genes [15, 18] has led to in silico inspection of harmful SNPs from huge datasets. In this context, genome sequencing investigations have detailed numerous genetic variants related to ADAM33; however, there are no comprehensive investigations for identifying damaging mutations beyond the huge pool of variant databases. Meta-analysis of ADAM33 mutation in asthma by Li et al. has been the sole systematic study carried out in recent years [18]. In that research, only F +1 (rs511898), Q1 (rs612709), and T2 (rs2280090) polymorphisms had confirmed functional influences in case-control studies. However, some reported polymorphisms vary from one population to another [11, 17].

Nevertheless, the probable consequences of numerous other mutations have stayed obscure. In general, genetic investigations are labor-intensive, time-consuming, and costly, while bioinformatic studies offer superior intuition into the right pathway of empirical research and considerably diminish expense and time [53]. Therefore, the present research tried to recognize functionally imperative nsSNPs in ADAM33 proteins using a variety of bioinformatic tools.

The prediction and further validation of the most damaging SNPs can be performed by merging various biocomputational-based procedures. In the present research, the scrutiny with seven prediction tools named SIFT, PolyPhen-2, PROVEAN, PANTHER, SNAP2, Align GVGD, and PredictSNP was carried out to get a blueprint of pathogenic nsSNPs of the ADAM33 gene. Due to the dependency of every algorithm on discrete parameters, 31 nsSNPs were selected (Table 1) as highly hazardous, which were predicted by all SNP prediction tools for further evaluation.

In general, a protein's activity, regulation, and function considerably rely on the stability of the protein molecule structure. Therefore, a reduction in the stability of protein leads to misfolding, degradation, and aggregation of proteins, resulting in posterior malfunction [54, 55]. For determination of the mentioned 31 deleterious nsSNPs' consequence on the ADAM33 protein stability, five online servers, including IMutant2.0, MutPred2, MUpro, NetSurfP.2, and SNPeffect, were used. Furthermore, SNPeffect, primarily the FoldX integrated tool with MutPred2 and MUpro, demonstrated a destabilizing impact upon mutations. At the same time, MutPred2 and NetSurfP.2 showed the effect of mutations on the function and structure of the desired protein. Therefore, the six SNPs leading to the reduction of protein stability, out of the 31 nsSNPs, could affect protein malfunction by consistency in the implemented tools.

The profile of evolutionary conservation of a protein contributes to ascertaining the harshness of a damaging mutation. The location of mutations in highly conserved regions is more likely to be harmful than mutations positioned in variable regions [56]. The ConSurf online tool was utilized to investigate the possible impacts of the 31 most detrimental nsSNPs (Table 6). This server provides data on evolutionary conservation along with predictions of solvent accessibility for locating putative functional and structural sites [57]. In addition, depending upon their location associated with the protein core or surface, extremely conserved residues are subordinated to be functional or structural, respectively [58]. Consistent with ConSurf, 17 damaging nsSNPs out of 31 possessed high conservation scores. Among these 17 highly conserved nsSNPs, 14 were forecasted as structural (buried), while the remaining were forecasted to be functional (exposed).

Prediction of structural and functional consequences of mutations on ADAM33 protein made up the central part of the present research; however, probing for the existence of damaging nsSNPs in the PTM and binding sites improves the heftiness of the obtained conclusion for the significance of that specific mutation. Various approaches have been employed to forecast ligand-binding sites, including structural templates, evolutionary data, and sequence conservation [59]. In the current research, the RaptorX server was recruited to predict the ligand-binding site of the desired protein sequence [35]. The domains recognized by RaptorX were in vote with those recognized by the InterPro server. The altered PTMs via SNPs may influence the protein structure and function; therefore, they can be considered biomarker nominees and drug targets for curative reasons [60].

Moreover, several researchers have reported that these alterations could considerably modify the protein's function by changing its stability, location, or interprotein interactions [7, 18, 61, 62]. A variety of PTMs were recognized based on the consensus motifs or chemical traits of amino acids. In this regard, all the software programs unanimously detected no mutation for the identified PTM sites.

Owing to the lack of human ADAM33 protein structure at the protein data bank, a protein modeling tool was applied to determine the 3D protein structure. The automated protein modeling tool called trROSETTA was used in which the whole protein FASTA sequence was an input file. The server offered five 5 top final structural models. The generated models demonstrated that D219V, C444Y, C388Y, C669G, C475G, and C606S mutants might result in noteworthy stereochemical aberration. Moreover, the quality of the forecasted models was validated via PROCHECK, Verify3D, ERRAT, and ProSAweb. Based on the default score of all validation tools, model 1 was chosen as the best tentative structure of the ADAM33 protein (Table 7). Finally, the TM-align tool was recruited for structural comparison among mutant and wild-type structures. High RMSD and low TM score values designated structural dissimilarity through C669G mutation, which showed a high RMSD value (3.0) and a relatively low TM score (0.586) [28, 55].

Before running the MD simulation, a preliminary analysis of structural stability alterations was performed using DynaMut and iMOD servers due to their advantages over other tools for stability change prediction. DynaMut identified D219V, C669G, C475G, and C606S as the most destabilizing mutations, whereas NMA results showed that only C475G and C444Y might cause structural stability in analogy to the wild-type protein. Therefore, the mutants D219V, C669G, and C606S were selected for MD simulation to detect the most deleterious mutations better. Literature research connected the stability of proteins with related atom fluctuations [63, 64]. In the current research, the coarse-grained (CG) simulation was carried out to determine its stability through the UNRES server. The analysis demonstrated that at a higher level of the radius of gyration, a decline in potential energy plot along with superior fluctuations was observed in all mutants in analogy to the wild-type protein (Figure 7). Therefore, it could be suggested that the mutations impact the stability and flexibility of the ADAM33 structure, perhaps thanks to global and local intramolecular perturbations. The findings of the dynamic simulations performed in this research agree with the protocol and results followed by Rodríguez-García et al. to evaluate the stability of the variations [65].

It has become imperative to perform gene prediction with particular DNA sequence polymorphisms by combining variant alleles and wild-type genotypes that influence vulnerability to a disease chiefly via interactions with environmental and genetic parameters [66]. In this context, GeneMANIA builds a complex gene-gene functional interaction network of the ADAM33 gene (Figure 3). The ADAM33 gene interaction network demonstrates that this gene has interacted most with other ADAM gene families. Moreover, ADAM33 has directly interacted with DGCR2, which encodes a new putative adhesion receptor protein that may have a function in neural crest cell migration [67]. On the other hand, ADAM33 interacts with CTSK and CTSV genes, phagosomal cathepsin genes, being related to lung diseases. These enzymes were reported to have a role in allergic airway inflammation [68, 69]. Therefore, damaging SNPs of the ADAM33 gene may influence the functioning and interaction of other genes involved in the gene-gene interaction network.

Furthermore, protein-protein interaction was also carried out using the STRING server to apprehend the functional interaction blueprint of the ADAM33 protein with other proteins within a cell. The findings demonstrated a strong interaction network with FCER1A, GSDMB, PHF11, NPSR1, and MS4A2 proteins involved in immune regulation and asthma disease [70–73]. Thus, the protein-protein interaction network of ADAM33 portrays attention to the influences of ADAM33 mutations which could impact other proteins implicated in asthma disease.

## 5. Conclusion

The exact biological role of ADAM33 protein has not been well understood; however, it has been proposed that the ADAM33 protein may have a potential role in the remodeling of the airway owing to its expression in myofibroblasts, epithelium, and ASMCs as well as its function in furthering stimulation of cell differentiation and proliferation along with angiogenesis. Moreover, ADAM33 may intervene in airway inflammation induced by environmental exposure and remodeling, likely via the TGF*β* signaling and various central receptors (AhR, TLRs, and CLRs). Hence, ADAM33 delineates a candidate target for asthma, and nsSNPs of this protein may influence asthma susceptibility. In the present research, 31 of 1055 nsSNPs of the human ADAM33 gene were forecasted as deleterious mutations using diverse computational software programs. More structural analysis disclosed that 6 SNPs, including D219V, C444Y, C388Y, C669G, C475G, and C606S, considerably influence ADAM33 protein stability. In additional evaluations using DynaMut and iMOD servers, D219V, C669G, and C606S were the most destabilizing SNPs between the six recognized mutations. Coarse-grained (CG) MD simulations were also carried out to explore how these mutations impact the protein structure. Simulation findings disclosed various considerable structural modifications, principally for the C669G variant, which significantly leads to the loss of hydrogen and disulfide bonds in the EGF-like domain.

Interestingly, this is the first systematic study of in silico evaluation of functional and structural nsSNPs in the ADAM33 protein. However, more clinical studies in various ethnic populations should be inspected in the future to confirm the outcomes of this evaluation. Furthermore, functional and structural inspections are also required to be performed in order to clarify the plausible mechanisms underlying the relation between nsSNPs and susceptibility to asthma disease.

## Figures and Tables

**Table 1:** Unanimous prediction of deleterious missense SNPs in the ADAM33 protein by different online software.

SNP ID	AAS	SIFT	PolyPhen-2	PANTHER-PSEP	SNPs&GO	PROVEAN	GVGD	PredictSNP
rs773091023	P678L	Deleterious	Probably damaging	Probably damaging	Disease	Deleterious	C65	Disease
rs764301730	C671R	Deleterious	Probably damaging	Probably damaging	Disease	Deleterious	C65	Disease
rs751597707	C669G	Deleterious	Probably damaging	Probably damaging	Disease	Deleterious	C65	Disease
rs368984072	G661R	Deleterious	Probably damaging	Probably damaging	Disease	Deleterious	C65	Disease
rs777553708	C637Y	Deleterious	Probably damaging	Probably damaging	Disease	Deleterious	C65	Disease
rs1334863089	C631F	Deleterious	Probably damaging	Probably damaging	Disease	Deleterious	C65	Disease
rs754867141	C606S	Deleterious	Probably damaging	Probably damaging	Disease	Deleterious	C65	Disease
rs754867141	C606Y	Deleterious	Probably damaging	Probably damaging	Disease	Deleterious	C65	Disease
rs915448220	C573Y	Deleterious	Probably damaging	Probably damaging	Disease	Deleterious	C65	Disease
rs1432549895	C519R	Deleterious	Probably damaging	Probably damaging	Disease	Deleterious	C65	Disease
rs1262246273	Y513C	Deleterious	Probably damaging	Probably damaging	Disease	Deleterious	C65	Disease
rs1203116461	P496L	Deleterious	Probably damaging	Probably damaging	Disease	Deleterious	C65	Disease
rs757567846	D483A	Deleterious	Probably damaging	Probably damaging	Disease	Deleterious	C65	Disease
rs1424975479	C482F	Deleterious	Probably damaging	Probably damaging	Disease	Deleterious	C65	Disease
rs1165412077	C482S	Deleterious	Probably damaging	Probably damaging	Disease	Deleterious	C65	Disease
rs779669835	R476C	Deleterious	Probably damaging	Probably damaging	Disease	Deleterious	C65	Disease
rs748720838	C475G	Deleterious	Probably damaging	Probably damaging	Disease	Deleterious	C65	Disease
rs528261077	G472V	Deleterious	Probably damaging	Probably damaging	Disease	Deleterious	C65	Disease
rs1378161696	C463Y	Deleterious	Probably damaging	Probably damaging	Disease	Deleterious	C65	Disease
rs768654352	G460R	Deleterious	Probably damaging	Probably damaging	Disease	Deleterious	C65	Disease
rs1192357091	C444S	Deleterious	Probably damaging	Probably damaging	Disease	Deleterious	C65	Disease
rs1192357091	C444Y	Deleterious	Probably damaging	Probably damaging	Disease	Deleterious	C65	Disease
rs777252478	C439Y	Deleterious	Probably damaging	Probably damaging	Disease	Deleterious	C65	Disease
rs1321728445	C431S	Deleterious	Probably damaging	Probably damaging	Disease	Deleterious	C65	Disease
rs747255442	G428C	Deleterious	Probably damaging	Probably damaging	Disease	Deleterious	C65	Disease
rs776082098	C420Y	Deleterious	Probably damaging	Probably damaging	Disease	Deleterious	C65	Disease
rs751613984	C388Y	Deleterious	Probably damaging	Probably damaging	Disease	Deleterious	C65	Disease
rs1313175203	C371Y	Deleterious	Probably damaging	Probably damaging	Disease	Deleterious	C65	Disease
rs764090883	C371R	Deleterious	Probably damaging	Probably damaging	Disease	Deleterious	C65	Disease
rs1297854732	C360R	Deleterious	Probably damaging	Probably damaging	Disease	Deleterious	C65	Disease
rs1351880201	D219V	Deleterious	Probably damaging	Probably damaging	Disease	Deleterious	C65	Disease

**Table 2:** Structural evaluations of detrimental mutants of ADAM33 protein by different online software.

SNP ID	AAS	MutPred2 Score^1^	I-MUTANT2.0		MuPro	
			Stability	RI^2^	Stability	delG^3^
rs773091023	P678L	0.64	Decreased	5	Increased	0.11
rs764301730	C671R	0.93	Decreased	5	Decreased	-1
rs751597707	C669G	0.89	Decreased	8	Decreased	-2.02
rs368984072	G661R	0.84	Decreased	7	Decreased	-0.62
rs777553708	C637Y	0.62	Decreased	3	Decreased	-0.65
rs1334863089	C631F	0.91	Decreased	3	Decreased	-0.06
rs754867141	C606S	0.93	Decreased	7	Decreased	-1.47
rs754867141	C606Y	0.93	Decreased	3	Decreased	-1
rs915448220	C573Y	0.93	Increased	1	Decreased	-0.75
rs1432549895	C519R	0.93	Decreased	2	Decreased	-0.93
rs1262246273	Y513C	0.93	Decreased	6	Decreased	-1.14
rs1203116461	P496L	0.84	Decreased	8	Decreased	-0.23
rs757567846	D483A	0.84	Decreased	4	Decreased	-1.01
rs1424975479	C482F	0.93	Decreased	5	Decreased	-0.51
rs1165412077	C482S	0.93	Decreased	6	Decreased	-1.22
rs779669835	R476C	0.92	Decreased	4	Decreased	-0.33
rs748720838	C475G	0.92	Decreased	7	Decreased	-1.66
rs528261077	G472V	0.92	Decreased	3	Decreased	-0.07
rs1378161696	C463Y	0.92	Decreased	2	Decreased	-0.77
rs768654352	G460R	0.87	Decreased	7	Decreased	-0.63
rs1192357091	C444S	0.92	Decreased	6	Decreased	-1.29
rs1192357091	C444Y	0.90	Decreased	4	Decreased	-0.84
rs777252478	C439Y	0.92	Decreased	2	Decreased	-0.56
rs1321728445	C431S	0.92	Decreased	5	Decreased	-1.46
rs747255442	G428C	0.92	Decreased	4	Decreased	-0.12
rs776082098	C420Y	0.93	Decreased	3	Decreased	-1.05
rs751613984	C388Y	0.93	Decreased	4	Decreased	-0.95
rs1313175203	C371Y	0.64	Decreased	0	Decreased	-0.83
rs764090883	C371R	0.78	Decreased	2	Decreased	-0.8
rs1297854732	C360R	0.91	Decreased	5	Decreased	-1.06
rs1351880201	D219V	0.92	Decreased	3	Decreased	-0.62

^1^Scores higher than 0.5 indicate pathogenicity. ^2^RI stands for reliability index of prediction. ^3^delG values under zero designate protein destabilization.

**Table 3:** Structural evaluations of detrimental mutants of ADAM33 protein by SNPeffect 4.0 web server.

SNP ID	AAS	TANGO	WALTS	LIMBO	FoldX
rs773091023	P678L	No effect	No effect	No effect	Not available
rs764301730	C671R	No effect	No effect	No effect	Not available
rs751597707	C669G	No effect	No effect	No effect	Not available
rs368984072	G661R	No effect	No effect	No effect	Not available
rs777553708	C637Y	No effect	No effect	No effect	Not available
rs1334863089	C631F	No effect	No effect	No effect	Not available
rs754867141	C606S	No effect	No effect	No effect	Not available
rs754867141	C606Y	No effect	No effect	No effect	Not available
rs915448220	C573Y	No effect	No effect	No effect	Not available
rs1432549895	C519R	No effect	No effect	No effect	Not available
rs1262246273	Y513C	No effect	No effect	No effect	Not available
rs1203116461	P496L	No effect	No effect	No effect	Not available
rs757567846	D483A	No effect	No effect	No effect	Not available
rs1424975479	C482F	No effect	No effect	No effect	Not available
rs1165412077	C482S	No effect	No effect	No effect	Not available
rs779669835	R476C	No effect	No effect	No effect	Not available
rs748720838	C475G	No effect	No effect	No effect	Not available
rs528261077	G472V	No effect	No effect	No effect	Not available
rs1378161696	C463Y	No effect	No effect	No effect	Not available
rs768654352	G460R	No effect	No effect	No effect	Not available
rs1192357091	C444S	No effect	No effect	No effect	Not available
rs1192357091	C444Y	No effect	Increases amyloid propensity	No effect	Not available
rs777252478	C439Y	No effect	No effect	No effect	Not available
rs747255442	G428C	No effect	No effect	No effect	Not available
rs747255442	G428C	No effect	No effect	No effect	Not available
rs776082098	C420Y	No effect	No effect	No effect	Not available
rs751613984	C388Y	No effect	Increases amyloid propensity	No effect	Reduce stability
rs1313175203	C371Y	No effect	No effect	No effect	Severely reduce stability
rs764090883	C371R	No effect	No effect	No effect	Severely reduce stability
rs1297854732	C360R	No effect	No effect	No effect	Reduce stability
rs1351880201	D219V	Increased aggregation tendency	Decreases amyloid propensity	No effect	Severely reduce stability

**Table 4:** Structural evaluations of detrimental mutants of ADAM33 protein by NetSurfP.2 web server.

SNP ID	AAS	NetSurfP.2					
		Wild class			Mutant class		
		RSA	ASA (A°)	Class alignment	Class alignment	RSA	ASA (A°)
rs773091023	P678L	24	33	Buried	Exposed	29	54
rs764301730	C671R	12	16	Buried	Buried	20	47
rs751597707	C669G	7	10	Buried	Buried	8	6
rs368984072	G661R	6	5	Buried	Buried	16	36
rs777553708	C637Y	3	4	Buried	Buried	4	9
rs1334863089	C631F	6	9	Buried	Buried	6	12
rs754867141	C606S	1	1	Buried	Buried	1	1
rs754867141	C606Y	1	1	Buried	Buried	1	3
rs915448220	C573Y	8	11	Buried	Buried	14	30
rs1432549895	C519R	12	17	Buried	Buried	20	47
rs1262246273	Y513C	24	51	Buried	Buried	16	22
rs1203116461	P496L	13	18	Buried	Buried	19	29
rs757567846	D483A	23	33	Buried	Buried	17	19
rs1424975479	C482F	10	14	Buried	Buried	14	28
rs1165412077	C482S	10	14	Buried	Buried	19	22
rs779669835	R476C	23	52	Buried	Buried	18	25
rs748720838	C475G	7	10	Buried	Buried	13	10
rs528261077	G472V	51	40	Exposed	Exposed	48	74
rs1378161696	C463Y	11	15	Buried	Buried	16	35
rs768654352	G460R	30	23	Exposed	Exposed	42	97
rs1192357091	C444S	8	11	Buried	Buried	17	20
rs1192357091	C444Y	8	11	Buried	Buried	11	23
rs777252478	C439Y	7	10	Buried	Buried	13	27
rs1321728445	C431S	5	7	Buried	Buried	8	10
rs747255442	G428C	70	55	Exposed	Exposed	61	85
rs776082098	C420Y	3	5	Buried	Buried	7	15
rs751613984	C388Y	8	12	Buried	Buried	11	24
rs1313175203	C371Y	4	6	Buried	Buried	9	19
rs764090883	C371R	4	6	Buried	Buried	12	28
rs1297854732	C360R	15	21	Buried	Exposed	50	113
rs1351880201	D219V	7	10	Buried	Buried	5	7

RSA stands for relative solvent accessibility. ASA stands for absolute solvent accessibility.

**Table 5:** Ligand-binding site prediction of ADAM33 protein, obtained from RaptorX binding server.

Domain	*P* value	uGDT (GDT)	uSeqId (SeqId)	Pocket	Multiplicity	Ligand	Binding residues
1 (residues 199-414)	4.79*e* − 09	4.79*e* − 09	205 (95)	1	150	ZN	A309 T310 V311 G312 L313 T342 H345 E346 H349 H355 A374 A375 A376 T377
				2	71	CA	E213 D296 N407

2 (residues 69-198)	7.79*e* − 03	52 (40)	9 (7)	1	9	PO4	E170 Q171 L172 L173 T174
				2	9	KDO	L172 L173 T174 W175 K176
				3	6	FTT	G135 S137 Y149 R151 E165 F167 M169 E170 Q171
				4	6	FTT	W127 S137 S147 Y149 M169 L172
				5	5	OES	P99 Q102 P103 Q117 R119
				6	3	OES	R121 P124 D125 S143

3 (residues 1-68)	2.69*e* − 02	23 (34)	5 (7)	1	1	7OD	L15 L19 T41 P42

4 (residues 415-690)	7.54*e* − 21	182 (66)	97 (35)	1	16	CA	L484 E486 D498 V499
				2	15	CA	L419 N422 F424 E426 E429 D432
				3	5	MAN	G480 D481

ZN: zinc ion; CA: calcium ion; PO_4_: phosphate ion; KDO: 3-deoxy-alpha-D-manno-oct-2-ulopyranosonic acid; FTT: 3-hydroxy-tetradecanoic acid; OES: N-octyl-2-hydroxyethyl sulfoxide; 7OD: (2E,5R)-5-hydroxy-2-methylhept-2-enoic acid; MAN: alpha-D-mannopyranose.

**Table 6:** Prediction of phylogenetic conservation in ADAM33 protein using ConSurf server.

SNP ID	AAS	Conservation score	Buried/exposed	Structural/functional
rs773091023	P678L	7	Exposed	—
rs764301730	C671R	8	Buried	—
rs751597707	C669G	8	Buried	—
rs368984072	G661R	8	Exposed	Functional
rs777553708	C637Y	8	Buried	—
rs1334863089	C631F	8	Exposed	Functional
rs754867141	C606S	8	Buried	—
rs754867141	C606Y	8	Buried	—
rs915448220	C573Y	9	Buried	Structural
rs1432549895	C519R	9	Buried	Structural
rs1262246273	Y513C	7	Exposed	—
rs1203116461	P496L	8	Exposed	Functional
rs757567846	D483A	9	Exposed	Functional
rs1424975479	C482F	9	Buried	Structural
rs1165412077	C482S	9	Buried	Structural
rs779669835	R476C	9	Exposed	Functional
rs748720838	C475G	9	Buried	Structural
rs528261077	G472V	8	Exposed	Functional
rs1378161696	C463Y	9	Buried	Structural
rs768654352	G460R	8	Exposed	Functional
rs1192357091	C444S	9	Buried	Structural
rs1192357091	C444Y	9	Buried	Structural
rs777252478	C439Y	9	Buried	Structural
rs1321728445	C431S	9	Buried	Structural
rs747255442	G428C	8	Exposed	Functional
rs776082098	C420Y	8	Buried	—
rs751613984	C388Y	9	Buried	Structural
rs1313175203	C371Y	9	Buried	Structural
rs764090883	C371R	9	Buried	Structural
rs1297854732	C360R	9	Buried	Structural
rs1351880201	D219V	9	Exposed	Functional

“b” indicates buried residue, “e” indicates exposed residue, “f” indicates functional residue (highly conserved and exposed), and “s” indicates structural residue (highly conserved and buried).

**Table 7:** Validation results of 3D modeled ADAM33 protein and its variants using different tools.

Model name	ProSAweb *Z* score	Verify3D residues with 3D-1D score ≥ 0.2	ERRAT quality factor	PROCHECK			
				Most favored regions	Additional allowed regions	Generously allowed regions	Disallowed regions
ADAM33	-9.53	97.59%	95.09	86.0%	12.8%	0.5%	0.7%
D219V	-9.43	97.99%	93.03	88.2%	10.1%	0.7%	1.0%
C388Y	-9.54	95.98%	91.83	86.0%	12.0%	1.0%	1.0%
C444Y	-9.40	95.18%	88.13	83.8%	12.5%	2.0%	1.7%
C475G	-9.77	95.18%	93.87	87.9%	11.3%	0.5%	0.2%
C606S	-9.53	98.19%	92.85	86.5%	11.8%	0.7%	1.0%
C669G	-9.31	92.77%	92.54	86.5%	11.1%	0.5%	2.0%

**Table 8:** TM-alignment: TM score and RMSD (Å) value for the high-risk nsSNPs in ADAM33 protein calculated using a TM-align calculator.

ASA	TM score	RMSD (Å)
D219V	0.979	1.30
C388Y	0.971	1.43
C444Y	0.565	2.21
C475G	0.569	2.00
C606S	0.970	1.68
C669G	0.586	3.00

**Table 9:** Prediction of changes in protein stability by DynaMut server.

#	AA from	AA to	Position	Prediction ΔΔ*G* ENCoM	ΔΔ*S* ENCoM	ΔΔ*G* DynaMut
1	C	S	606	-0.322 kcal/mol	0.402 kcal·mol^−1^·K^−1^	-0.571 kcal/mol
2	C	G	475	-0.416 kcal/mol	0.519 kcal·mol^−1^·K^−1^	-0.457 kcal/mol
3	C	Y	444	0.202 kcal/mol	-0.252 kcal·mol^−1^·K^−1^	0.645 kcal/mol
4	C	G	669	-0.67 kcal/mol	0.838 kcal·mol^−1^·K^−1^	-0.948 kcal/mol
5	C	Y	388	0.341 kcal/mol	-0.426 kcal·mol^−1^·K^−1^	0.299 kcal/mol
6	C	R	360	0.383 kcal/mol	-0.478 kcal·mol^−1^·K^−1^	0.574 kcal/mol
7	D	V	219	0.07 kcal/mol	-0.088 kcal·mol^−1^·K^−1^	-0.457 kcal/mol

## Data Availability

All data used to support the findings of this study are included within the supplementary information files.

## Supplementary Materials

Supplementary Materials Table S1 provides information about which amino acid substitution may alter the molecular mechanism of the ADAM33 enzyme. Tables S2-S4 provide information on possible posttranslational modifications of amino acid substitution within the ADAM33 enzyme. Finally, Figures S1-S7 portray the normal mode analysis of wild-type and mutated proteins including *B*-factor, deformability, eigenvalue, variance, and elastic network.
