# Supplementary material for: In Silico Evaluation of Nonsynonymous SNPs in Human ADAM33: The Most Common Form of Genetic Association to Asthma Susceptibility
Source: Comput Math Methods Med. 2022 Nov 12;2022:1089722. doi: 10.1155/2022/1089722 (PMC9675607; doi:10.1155/2022/1089722)
Supplement: Supplementary Materials — Table S1 provides information about which amino acid substitution may alter the molecular mechanism of the ADAM33 enzyme. Tables S2-S4 provide information on possible posttranslational modifications of amino acid substitution within the ADAM33 enzyme. Finally, Figures S1-S7 portray the normal mode analysis of wild-type and mutated proteins including B-factor, deformability, eigenvalue, variance, and elastic network. [file 1089722.f1.zip › Legend for Figure S1-S7.pdf]

Figure S1: The results of normal mode analysis of ADAM33 proteins. Here, (a) deformability, (b) B-factor, (c) eigenvalues, (d) variance (red color indicates individual variances and green color indicates cumulative variances), (e) co-variance map (correlated (red), uncorrelated (white) or anti-correlated (blue) motions), and (f) elastic network (darker gray regions designate more stiffer regions)

Figure S2: The results of normal mode analysis of D219V mutated proteins. Here, (a) deformability, (b) B-factor, (c) eigenvalues, (d) variance (red color indicates individual variances and green color indicates cumulative variances), (e) co-variance map (correlated (red), uncorrelated (white) or anti-correlated (blue) motions), and (f) elastic network (darker gray regions designate more stiffer regions)

Figure S3: The results of normal mode analysis of C388Y mutated proteins. Here, (a) deformability, (b) B-factor, (c) eigenvalues, (d) variance (red color indicates individual variances and green color indicates cumulative variances), (e) co-variance map (correlated (red), uncorrelated (white) or anti-correlated (blue) motions), and (f) elastic network (darker gray regions designate more stiffer regions)

Figure S4: The results of normal mode analysis of C444Y mutated proteins. Here, (a) deformability, (b) B-factor, (c) eigenvalues, (d) variance (red color indicates individual variances and green color indicates cumulative variances), (e) co-variance map (correlated (red), uncorrelated (white) or anti-correlated (blue) motions), and (f) elastic network (darker gray regions designate more stiffer regions)

Figure S5: The results of normal mode analysis of C475G mutated proteins. Here, (a) deformability, (b) B-factor, (c) eigenvalues, (d) variance (red color indicates individual variances and green color indicates cumulative variances), (e) co-variance map (correlated (red), uncorrelated (white) or anti-correlated (blue) motions), and (f) elastic network (darker gray regions designate more stiffer regions)

Figure S6: The results of normal mode analysis of C606G mutated proteins. Here, (a) deformability, (b) B-factor, (c) eigenvalues, (d) variance (red color indicates individual variances and green color indicates cumulative variances), (e) co-variance map (correlated (red), uncorrelated (white) or anti-correlated (blue) motions), and (f) elastic network (darker gray regions designate more stiffer regions)

Figure S7: The results of normal mode analysis of C669G mutated proteins. Here, (a) deformability, (b) B-factor, (c) eigenvalues, (d) variance (red color indicates individual variances and green color indicates cumulative variances), (e) co-variance map (correlated (red), uncorrelated (white) or anti-correlated (blue) motions), and (f) elastic network (darker gray regions designate more stiffer regions)
