# Supplementary material for: In Silico Evaluation of Nonsynonymous SNPs in Human ADAM33: The Most Common Form of Genetic Association to Asthma Susceptibility
Source: Comput Math Methods Med. 2022 Nov 12;2022:1089722. doi: 10.1155/2022/1089722 (PMC9675607; doi:10.1155/2022/1089722)
Supplement: Supplementary Materials — Table S1 provides information about which amino acid substitution may alter the molecular mechanism of the ADAM33 enzyme. Tables S2-S4 provide information on possible posttranslational modifications of amino acid substitution within the ADAM33 enzyme. Finally, Figures S1-S7 portray the normal mode analysis of wild-type and mutated proteins including B-factor, deformability, eigenvalue, variance, and elastic network. [file 1089722.f1.zip › Table S1.pdf]

Table S1: The prediction outcomes for the nsSNPs effect on ADAM33 protein by MutPred server.

SNP ID	AAS	Probability of deleterious amino acid substitution	Top 5 features
rs773091023	P678L	0.800	Altered Transmembrane protein<br>Loss of Loop<br>Gain of Disulfide linkage at C680
rs764301730	C671R	0.951	Altered Metal binding<br>Altered Transmembrane protein<br>Gain of Catalytic site at C671<br>Loss of Disulfide linkage at C671
rs751597707	C669G	0.937	Altered Metal binding<br>Altered Transmembrane protein<br>Altered Stability<br>Gain of Disulfide linkage at C671<br>Gain of Catalytic site at C669
rs368984072	G661R	0.897	Altered Metal binding<br>Altered Transmembrane protein<br>Loss of Disulfide linkage at C663
rs777553708	C637Y	0.943	Altered Transmembrane protein<br>Loss of Disulfide linkage at C637<br>Altered Metal binding<br>Gain of Strand<br>Altered Disordered interface
rs1334863089	C631F	0.953	Altered Metal binding<br>Altered Transmembrane protein<br>Loss of Disulfide linkage at C631<br>Gain of Catalytic site at C631
rs754867141	C606S	0.857	Loss of Disulfide linkage at C606<br>Altered Metal binding<br>Altered Transmembrane protein<br>Altered Stability<br>Gain of O-linked glycosylation at T605<br>Gain of Catalytic site at C606
rs754867141	C606Y	0.915	Loss of Disulfide linkage at C606<br>Altered Metal binding<br>Altered Transmembrane protein<br>Gain of Catalytic site at C606
rs915448220	C573Y	0.953	Altered Metal binding<br>Altered Transmembrane protein<br>Gain of Strand<br>Gain of Catalytic site at C573<br>Loss of Disulfide linkage at C573
rs1432549895	C519R	0.940	Loss of Disulfide linkage at C519<br>Gain of Catalytic site at C519<br>Altered Transmembrane protein<br>Altered Metal binding<br>Loss of Pyrrolidone carboxylic acid at Q524
rs1262246273	Y513C	0.898	Altered Transmembrane protein<br>Gain of Intrinsic disorder<br>Loss of Disulfide linkage at C514<br>Loss of Strand<br>Altered Ordered interface
rs1203116461	P496L	0.668	Altered Transmembrane protein

			Gain of Helix<br>Loss of Strand<br>Loss of Loop<br>Altered Metal binding
rs757567846	D483A	0.839	Altered Metal binding<br>Gain of Disulfide linkage at C482<br>Altered Transmembrane protein
rs1424975479	C482F	0.900	Altered Metal binding<br>Loss of Disulfide linkage at C482<br>Altered Transmembrane protein<br>Loss of Pyrrolidone carboxylic acid at Q477
rs1165412077	C482S	0.794	Altered Metal binding<br>Loss of Disulfide linkage at C482<br>Altered Transmembrane protein<br>Loss of Pyrrolidone carboxylic acid at Q477<br>Gain of Catalytic site at C482
rs779669835	R476C	0.834	Altered Metal binding<br>Loss of Disulfide linkage at C475<br>Loss of Pyrrolidone carboxylic acid at Q477<br>Altered Transmembrane protein
rs748720838	C475G	0.942	Altered Metal binding<br>Loss of Disulfide linkage at C475<br>Loss of Pyrrolidone carboxylic acid at Q477<br>Altered Transmembrane protein
rs528261077	G472V	0.828	Altered Metal binding<br>Gain of Disulfide linkage at C475<br>Gain of Pyrrolidone carboxylic acid at Q477
rs1378161696	C463Y	0.915	Altered Metal binding<br>Loss of Intrinsic disorder<br>Altered Transmembrane protein<br>Gain of Disulfide linkage at C462
rs768654352	G460R	0.856	Gain of Helix<br>Altered Metal binding<br>Gain of Disulfide linkage at C457<br>Altered Transmembrane protein<br>Gain of Pyrrolidone carboxylic acid at Q456
rs1192357091	C444S	0.852	Altered Metal binding<br>Loss of Disulfide linkage at C444<br>Altered Transmembrane protein<br>Gain of N-linked glycosylation at N448
rs1192357091	C444Y	0.914	Altered Metal binding<br>Loss of Intrinsic disorder<br>Loss of Disulfide linkage at C444<br>Altered Transmembrane protein<br>Gain of N-linked glycosylation at N448
rs777252478	C439Y	0.790	Loss of Intrinsic disorder<br>Altered Metal binding<br>Loss of Disulfide linkage at C444
rs1321728445	C431S	0.881	Altered Metal binding<br>Gain of Strand<br>Gain of B-factor<br>Altered Transmembrane protein<br>Loss of Disulfide linkage at C431
rs747255442	G428C	0.813	Altered Metal binding<br>Altered Transmembrane protein

			Gain of Disulfide linkage at C433
rs776082098	C420Y	0.917	Loss of Intrinsic disorder<br>Altered Metal binding<br>Gain of Strand<br>Altered Transmembrane protein<br>Loss of Disulfide linkage at C420
rs751613984	C388Y	0.884	Altered Metal binding<br>Altered Disordered interface<br>Altered Transmembrane protein<br>Loss of Disulfide linkage at C388<br>Gain of Sulfation at C388
rs1313175203	C371Y	0.963	Altered Metal binding<br>Gain of Strand<br>Loss of Disulfide linkage at C371<br>Altered Transmembrane protein<br>Gain of Catalytic site at C371
rs764090883	C371R	0.971	Altered Metal binding<br>Gain of Intrinsic disorder<br>Gain of Strand<br>Loss of Disulfide linkage at C371<br>Altered Transmembrane protein
rs1297854732	C360R	0.882	Altered Metal binding<br>Gain of Strand<br>Loss of Disulfide linkage at C360<br>Gain of Allosteric site at H355<br>Loss of Catalytic site at H355
rs1351880201	D219V	0.9355	Altered Ordered interface<br>Altered Metal binding<br>Altered Transmembrane protein<br>Altered Disordered interface<br>Loss of Sulfation at Y215
