# Supplementary material for: In Silico Evaluation of Nonsynonymous SNPs in Human ADAM33: The Most Common Form of Genetic Association to Asthma Susceptibility
Source: Comput Math Methods Med. 2022 Nov 12;2022:1089722. doi: 10.1155/2022/1089722 (PMC9675607; doi:10.1155/2022/1089722)
Supplement: Supplementary Materials — Table S1 provides information about which amino acid substitution may alter the molecular mechanism of the ADAM33 enzyme. Tables S2-S4 provide information on possible posttranslational modifications of amino acid substitution within the ADAM33 enzyme. Finally, Figures S1-S7 portray the normal mode analysis of wild-type and mutated proteins including B-factor, deformability, eigenvalue, variance, and elastic network. [file 1089722.f1.zip › Table S2.pdf]

Table S2: Prediction of ubiquitination site in ADAM33 protein using BDM-PUB server

Peptide	Position	Score	Threshold
ELLLELEKNHRLLAP	83	0.63	0.3
PWPPRGSKDFSTHEI	159	1.41	0.3
MEQLLTWKGTCGHRD	176	0.65	0.3
GHRDPGNKAGMTSLP	187	0.96	0.3
REARRTRKYLELYIV	210	1.05	0.3
QLRAFFRKGGGACLS	399	1.49	0.3
CCVRCLLKPAGALCR	469	1.56	0.3
GRDALCGKLQCQGGK	575	1.66	0.3
KLQCQGGKPSLLAPH	582	0.98	0.3
CQSRRCRKNAFQELQ	644	1.65	0.3
WAPPFCDKPGFGGSM	682	0.68	0.3
DPACSGPKDGPHRDH	749	1.71	0.3
EPSSHPEKPLPAVSP	791	0.75	0.3
