# Supplementary material for: In Silico Evaluation of Nonsynonymous SNPs in Human ADAM33: The Most Common Form of Genetic Association to Asthma Susceptibility
Source: Comput Math Methods Med. 2022 Nov 12;2022:1089722. doi: 10.1155/2022/1089722 (PMC9675607; doi:10.1155/2022/1089722)
Supplement: Supplementary Materials — Table S1 provides information about which amino acid substitution may alter the molecular mechanism of the ADAM33 enzyme. Tables S2-S4 provide information on possible posttranslational modifications of amino acid substitution within the ADAM33 enzyme. Finally, Figures S1-S7 portray the normal mode analysis of wild-type and mutated proteins including B-factor, deformability, eigenvalue, variance, and elastic network. [file 1089722.f1.zip › Table S3.pdf]

Table S3: Prediction of phosphorylation sites in ADAM33 protein using NetPhos server

Amino Acid Position	Context	Score	Kinase
11 T	RARGTPLLL	0.957	unspecified
55 S	WRTVSLEEP	0.995	unspecified
61 S	EEPVSKPDM	0.964	unspecified
162 S	SKDFSTHEI	0.995	unspecified
269 S	ERDRSRVTQ	0.996	unspecified
302 T	AQLLTGRAF	0.902	Protein Kinase C
324 S	CRAESSGGV	0.920	unspecified
329 S	SGGVSTDHS	0.947	unspecified
511 S	CARGSGYCW	0.962	unspecified
547 S	QVVNSAGDA	0.988	unspecified
639 S	MVCQSRRCR	0.913	Protein Kinase C
787 S	HEPSSHPEK	0.992	unspecified
797 S	LPAVSPDPQ	0.909	unspecified
