# Supplementary material for: In Silico Evaluation of Nonsynonymous SNPs in Human ADAM33: The Most Common Form of Genetic Association to Asthma Susceptibility
Source: Comput Math Methods Med. 2022 Nov 12;2022:1089722. doi: 10.1155/2022/1089722 (PMC9675607; doi:10.1155/2022/1089722)
Supplement: Supplementary Materials — Table S1 provides information about which amino acid substitution may alter the molecular mechanism of the ADAM33 enzyme. Tables S2-S4 provide information on possible posttranslational modifications of amino acid substitution within the ADAM33 enzyme. Finally, Figures S1-S7 portray the normal mode analysis of wild-type and mutated proteins including B-factor, deformability, eigenvalue, variance, and elastic network. [file 1089722.f1.zip › Table S4.pdf]

N-linked Glycosites

Position	Residue Score	Prediction
84	-0.75738241	Non-glycosylated
109	**1.1133404**	**Potential Glycosylated**
145	**0.74524993**	**Potential Glycosylated**
186	-1.263588	Non-glycosylated
229	-0.96618467	Non-glycosylated
231	**0.93513567**	**Potential Glycosylated**
242	-0.9980678	Non-glycosylated
276	**1.1491922**	**Potential Glycosylated**
407	-1.0217595	Non-glycosylated
422	-1.2994016	Non-glycosylated
448	**0.85056617**	**Potential Glycosylated**
546	-1.2768468	Non-glycosylated
554	-1.0981906	Non-glycosylated
645	-1.0863686	Non-glycosylated
664	-1.1641786	Non-glycosylated
666	-1.2469535	Non-glycosylated
668	-1.212558	Non-glycosylated
698	-0.85489265	Non-glycosylated
781	-1.002133	Non-glycosylated

O-linked Glycosites

Position	Residue Score	Prediction
11	-0.008041936	Non-glycosylated
41	0.017683314	Potential Glycosylated
53	0.14775573	Potential Glycosylated
55	0.13899511	Potential Glycosylated
61	0.064735369	Potential Glycosylated
95	0.21011165	Potential Glycosylated
111	0.020847242	Potential Glycosylated
126	-0.07743013	Non-glycosylated
132	-0.11841244	Non-glycosylated
134	-0.16366568	Non-glycosylated
137	-0.2218519	Non-glycosylated
141	-0.14758465	Non-glycosylated
143	-0.16199966	Non-glycosylated
147	0.0208001	Potential Glycosylated
158	-0.052241223	Non-glycosylated
162	-0.042439932	Non-glycosylated
163	-0.22618827	Non-glycosylated
174	-0.1674848	Non-glycosylated
178	-0.17008351	Non-glycosylated
191	0.099266654	Potential Glycosylated
192	0.15203628	Potential Glycosylated
199	0.033640983	Potential Glycosylated
208	-0.041493815	Non-glycosylated
221	-0.065626414	Non-glycosylated
225	-0.089431455	Non-glycosylated
233	-0.01953224	Non-glycosylated
250	-0.0061406778	Non-glycosylated
258	-0.07450101	Non-glycosylated
264	-0.12088642	Non-glycosylated

C-linked Glycosites

Position	Residue Score	Prediction
3	-0.5510491	Non-glycosylated
23	-0.63565208	Non-glycosylated
44	-0.44351012	Non-glycosylated
51	-0.56757255	Non-glycosylated
127	-0.38798279	Non-glycosylated
153	-0.4564656	Non-glycosylated
175	-0.70791733	Non-glycosylated
263	-0.41029443	Non-glycosylate
280	-0.59604798	Non-glycosylated
285	-0.77052887	Non-glycosylated
290	-0.64643199	Non-glycosylated
515	-0.56108422	Non-glycosylated
530	-0.56807419	Non-glycosylated
675	-0.70722165	Non-glycosylated
722	-0.56299283	Non-glycosylated
737	-0.47025367	Non-glycosylated
775	-0.51115416	Non-glycosylated
813	-0.55151633	Non-glycosylated

269	-0.11950397	Non-glycosylated
272	0.0022645529	Potential Glycosylated
278	-0.021963579	Non-glycosylated
297	0.014469633	Potential Glycosylated
302	0.19336471	Potential Glycosylated
310	-0.06636103	Non-glycosylated
324	-0.0089418155	Non-glycosylated
325	-0.11577082	Non-glycosylated
329	-0.0038611931	Non-glycosylated
330	0.21967785	Potential Glycosylated
333	0.27408455	Potential Glycosylated
342	-0.014657802	Non-glycosylated
350	-0.090528209	Non-glycosylated
354	-0.087275927	Non-glycosylated
368	-0.0081473722	Non-glycosylated
377	0.05576549	Potential Glycosylated
386	-0.028509932	Non-glycosylated
389	-0.031181631	Non-glycosylated
406	-0.023705064	Non-glycosylated
450	-0.035364814	Non-glycosylated
489	0.011167192	Potential Glycosylated
491	0.17105456	Potential Glycosylated
492	0.27170437	Potential Glycosylated
493	0.32483205	Potential Glycosylated
505	-0.059114232	Non-glycosylated
511	-0.061842677	Non-glycosylated
521	-0.035476011	Non-glycosylated
534	-0.016521484	Non-glycosylated
547	-0.051190277	Non-glycosylated
559	-0.054381553	Non-glycosylated
584	-0.0037674937	Non-glycosylated

595	0.040512395	Potential Glycosylated
596	0.026744187	Potential Glycosylated
605	0.19823099	Potential Glycosylated
614	0.066121128	Potential Glycosylated
629	0.013426558	Potential Glycosylated
639	0.030352585	Potential Glycosylated
655	-0.025484912	Non-glycosylated
659	-0.089604904	Non-glycosylated
665	-0.034440488	Non-glycosylated
688	-0.048535275	Non-glycosylated
691	-0.069729603	Non-glycosylated
701	-0.0073701317	Non-glycosylated
709	0.0053411819	Potential Glycosylated
736	-0.069919075	Non-glycosylated
746	-0.043307183	Non-glycosylated
769	0.1283968	Potential Glycosylated
771	0.27119581	Potential Glycosylated
782	0.012007174	Potential Glycosylated
786	0.035984737	Potential Glycosylated
787	0.055670894	Potential Glycosylated
797	0.014707479	Potential Glycosylated
810	0.0057643317	Potential Glycosylated
