# Supplementary figures and images for: In Silico Evaluation of Nonsynonymous SNPs in Human ADAM33: The Most Common Form of Genetic Association to Asthma Susceptibility
Source: Comput Math Methods Med. 2022 Nov 12;2022:1089722. doi: 10.1155/2022/1089722 (PMC9675607; doi:10.1155/2022/1089722)

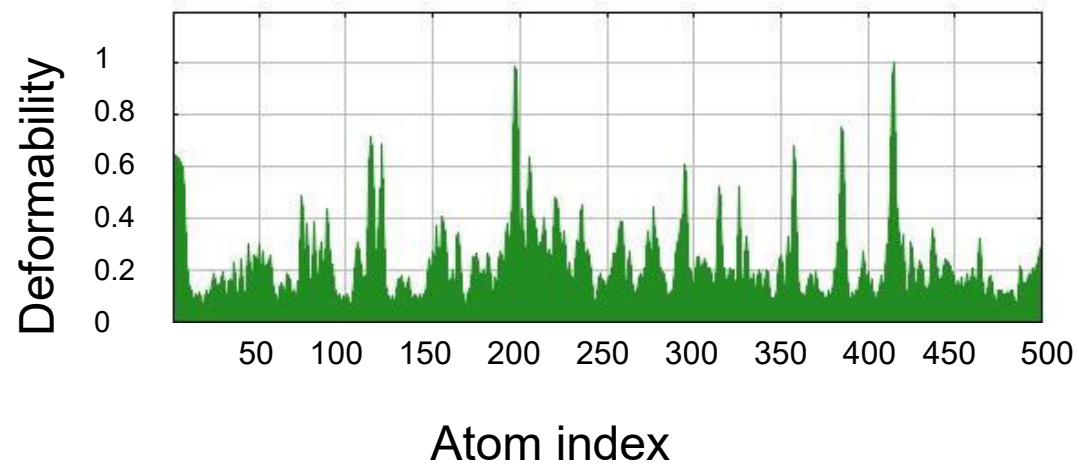

a

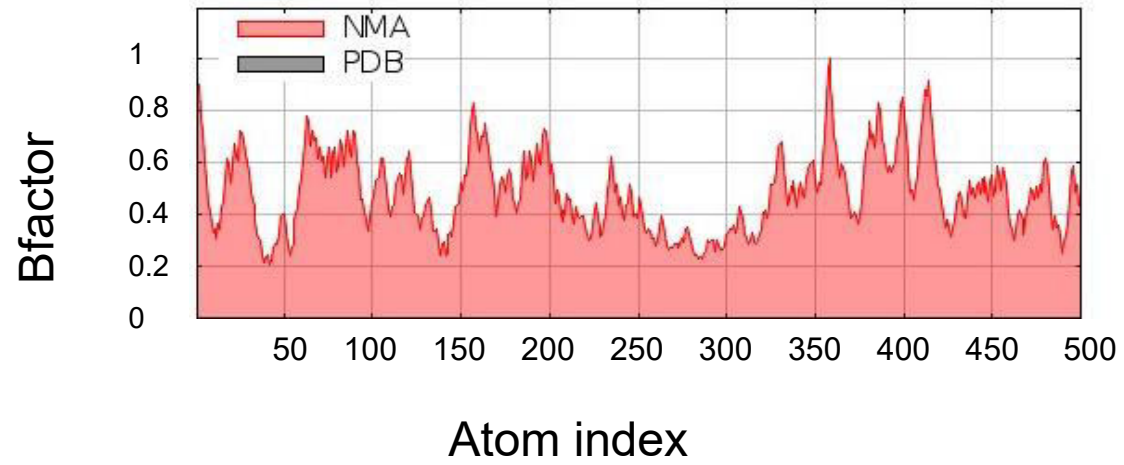

b

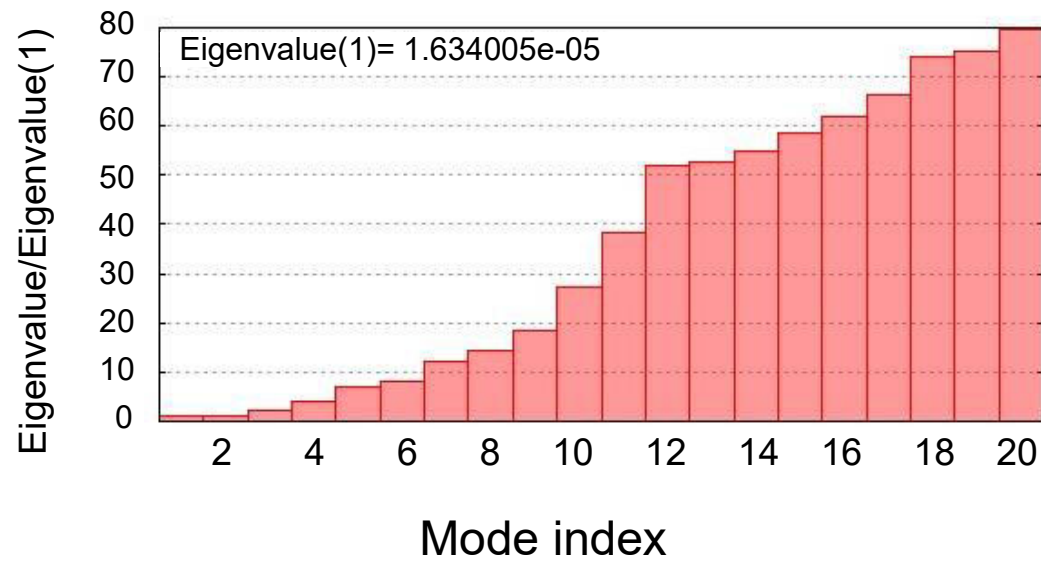

c

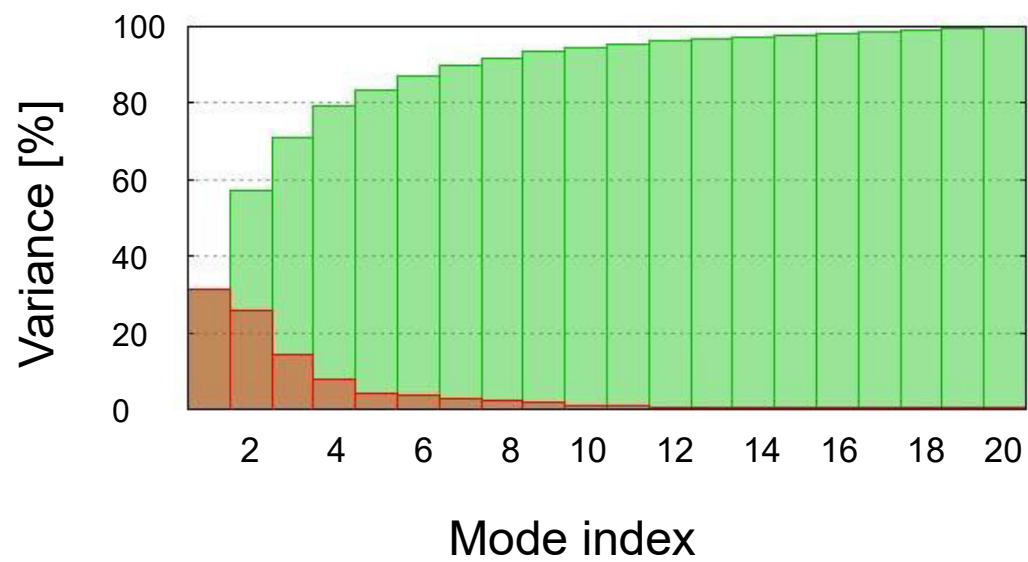

d

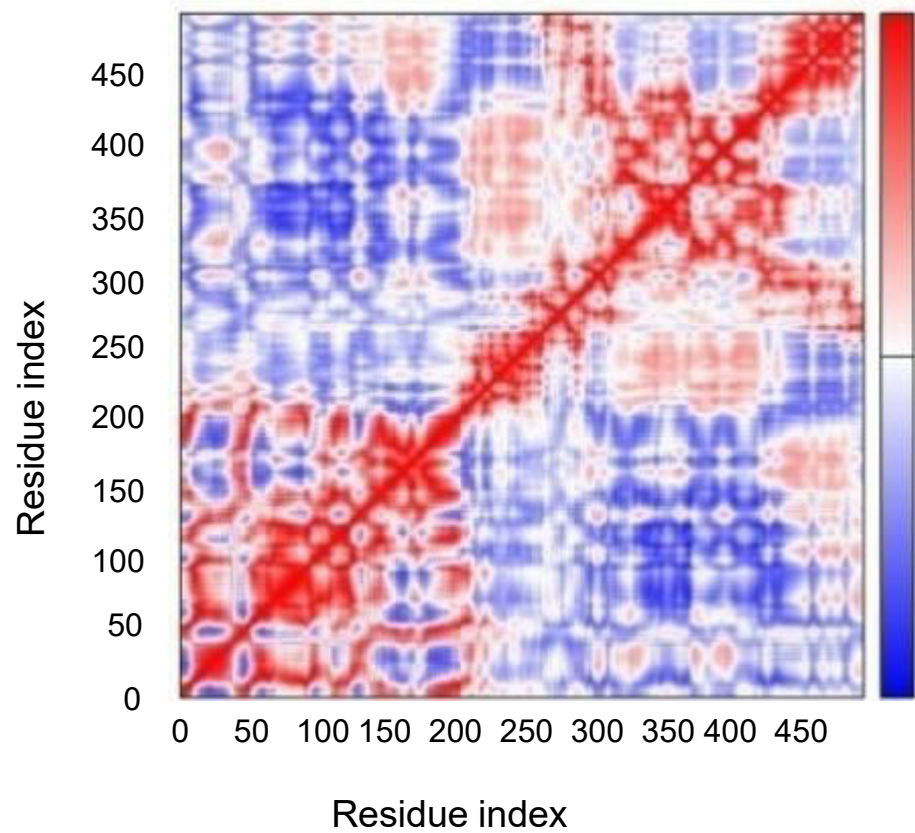

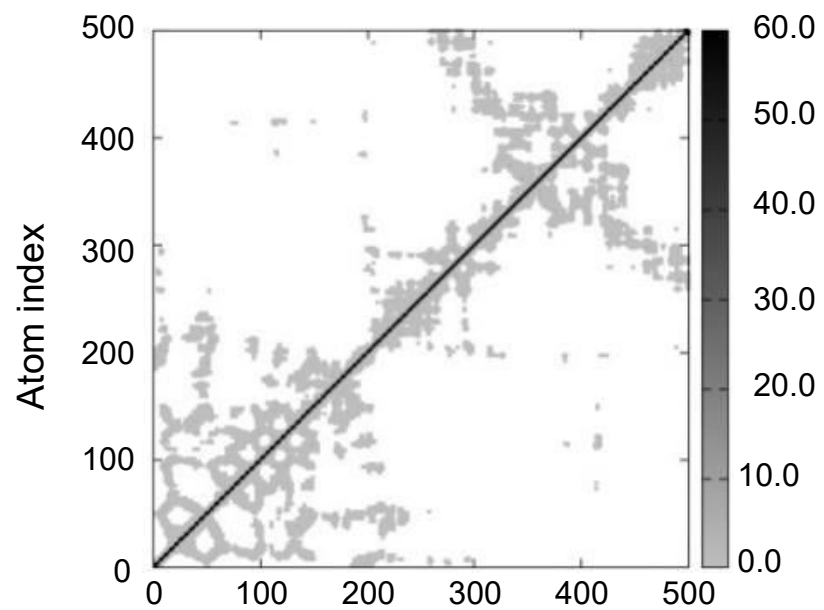

f

Atom index

Supplement: Supplementary Materials — Table S1 provides information about which amino acid substitution may alter the molecular mechanism of the ADAM33 enzyme. Tables S2-S4 provide information on possible posttranslational modifications of amino acid substitution within the ADAM33 enzyme. Finally, Figures S1-S7 portray the normal mode analysis of wild-type and mutated proteins including B-factor, deformability, eigenvalue, variance, and elastic network. [file 1089722.f1.zip › Figure S1 (1).pdf]

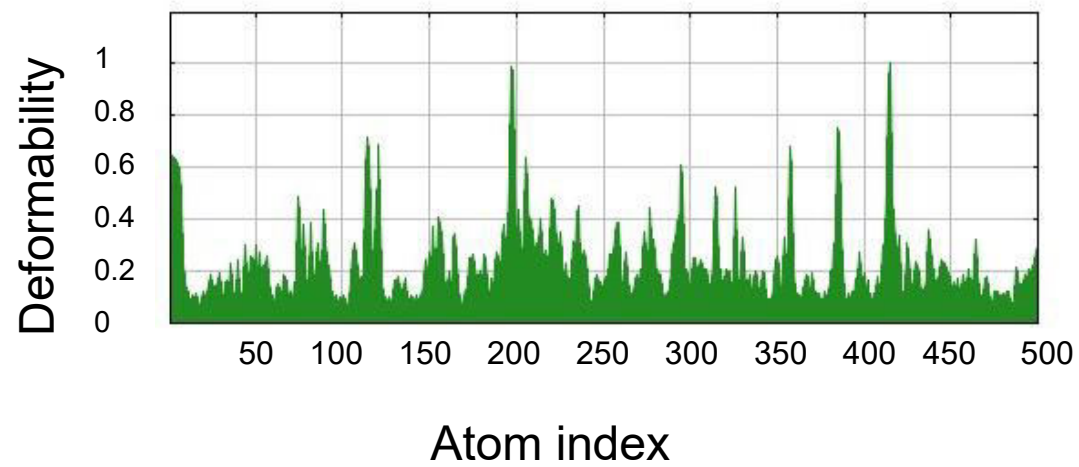

a

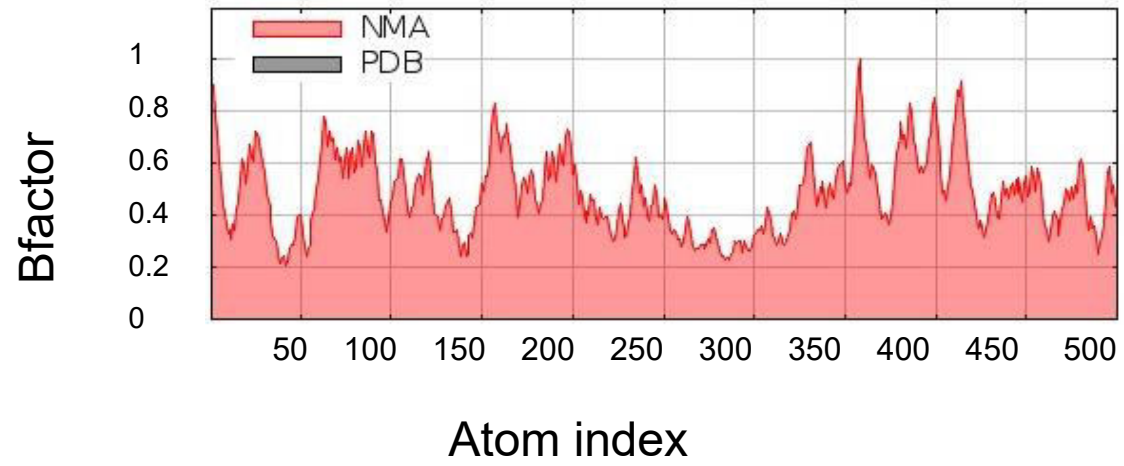

b

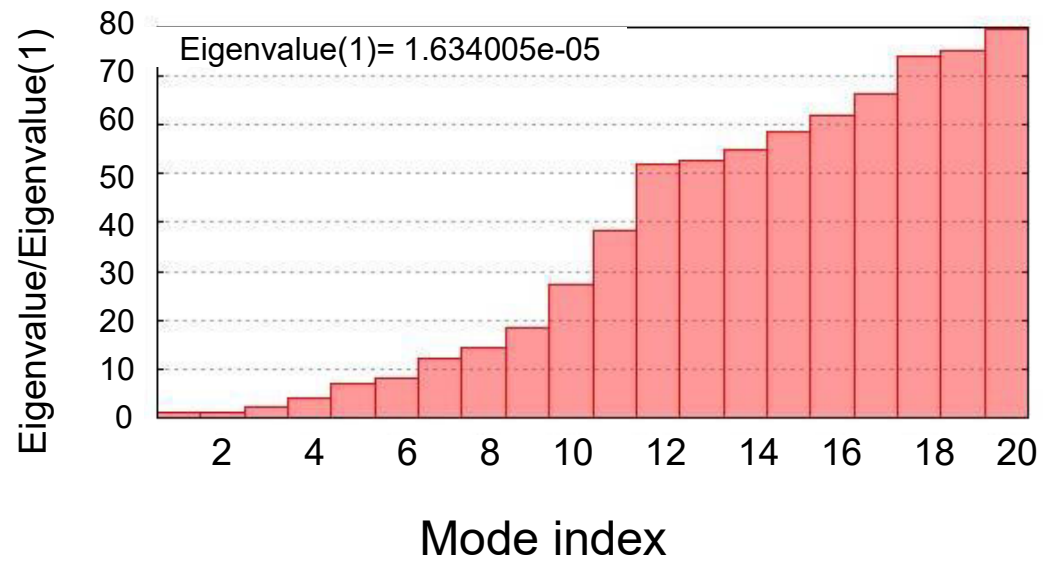

c

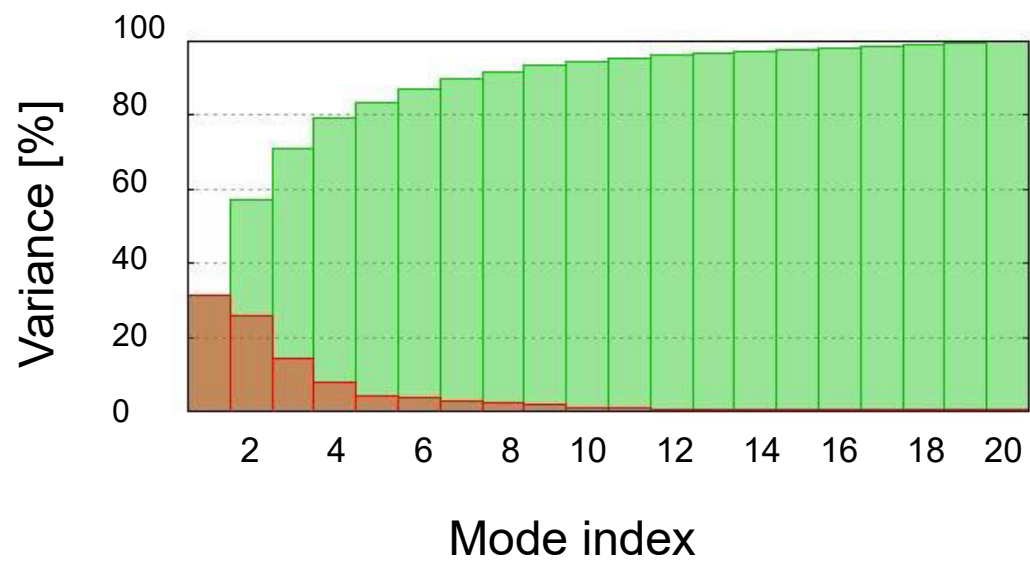

d

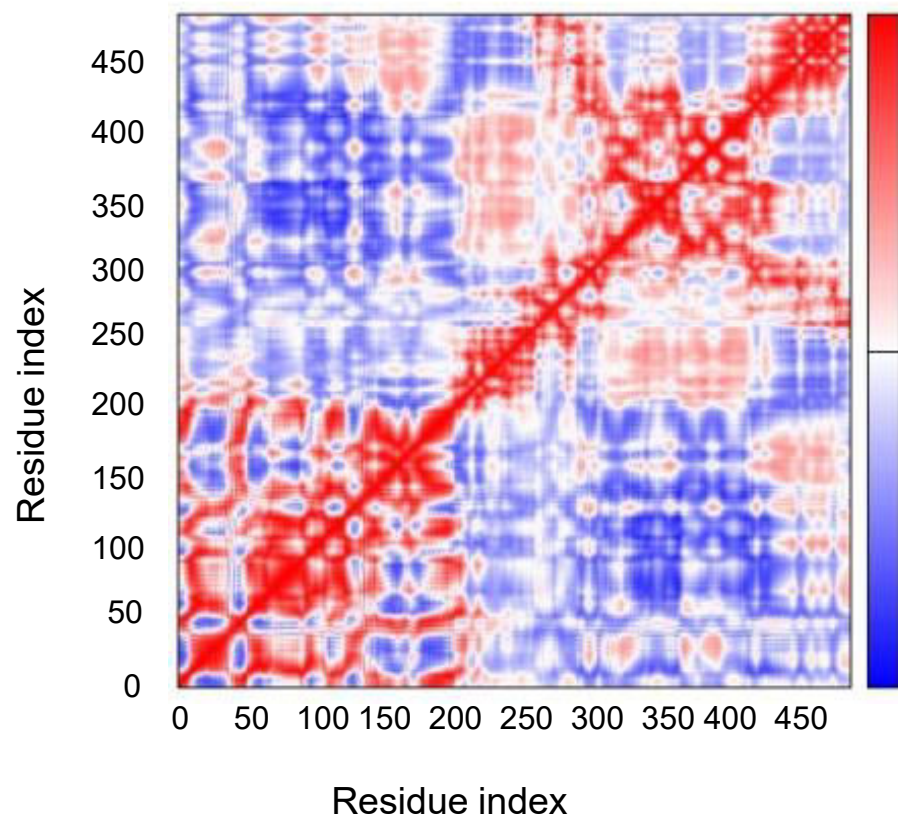

e

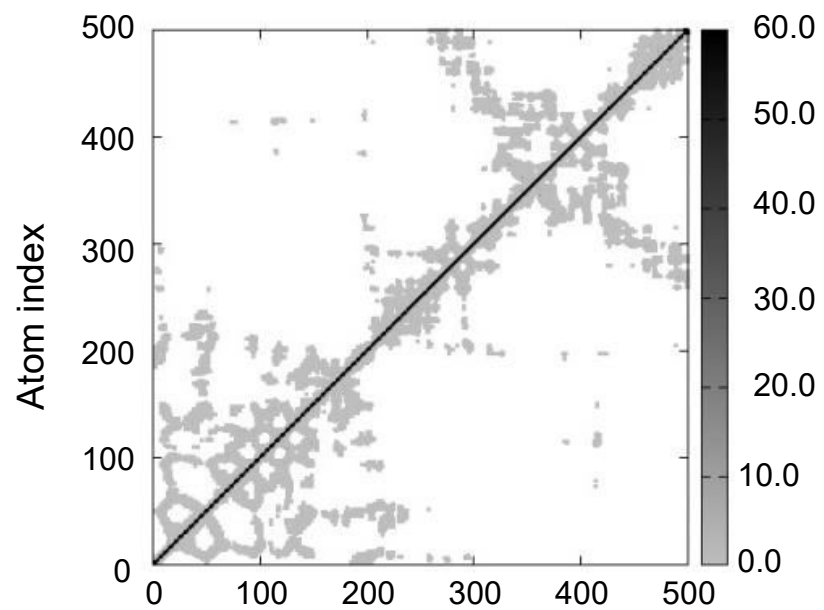

f

Atom index

Supplement: Supplementary Materials — Table S1 provides information about which amino acid substitution may alter the molecular mechanism of the ADAM33 enzyme. Tables S2-S4 provide information on possible posttranslational modifications of amino acid substitution within the ADAM33 enzyme. Finally, Figures S1-S7 portray the normal mode analysis of wild-type and mutated proteins including B-factor, deformability, eigenvalue, variance, and elastic network. [file 1089722.f1.zip › Figure S2.pdf]

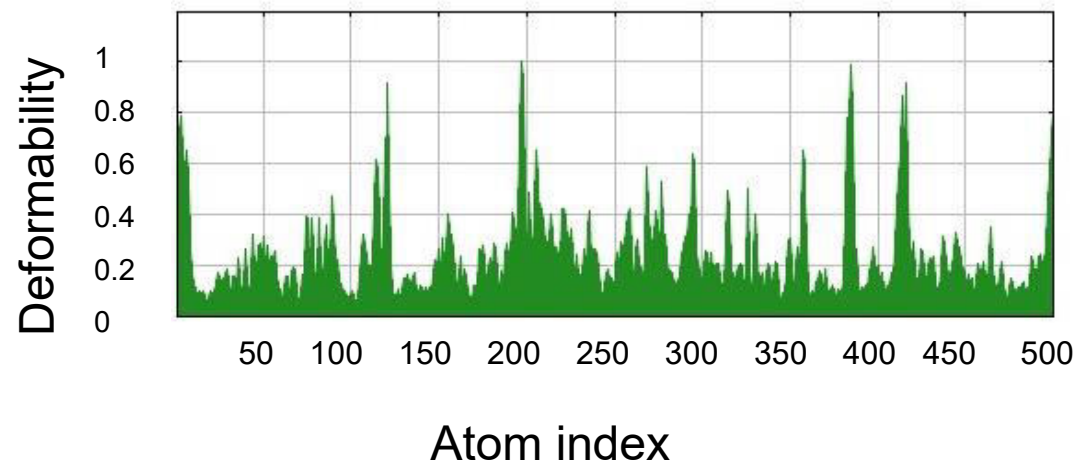

a

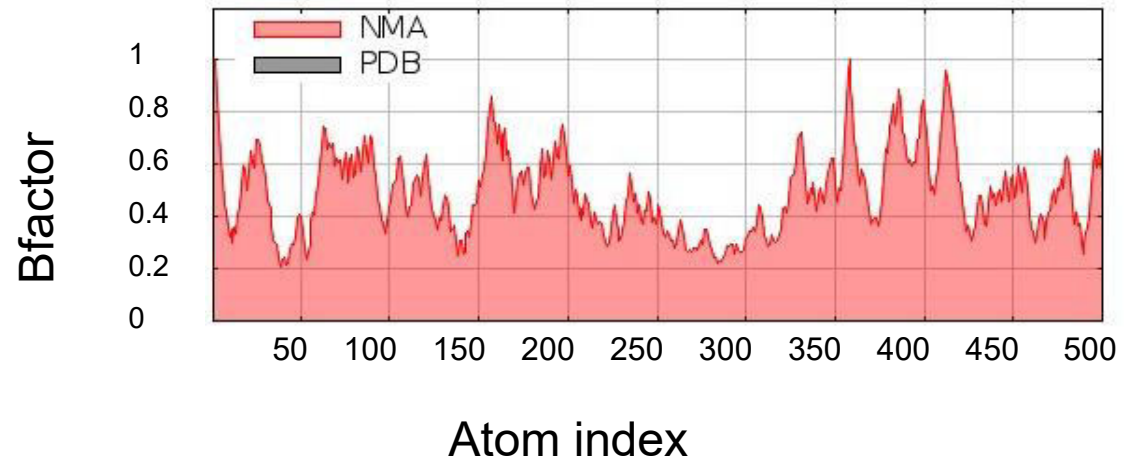

b

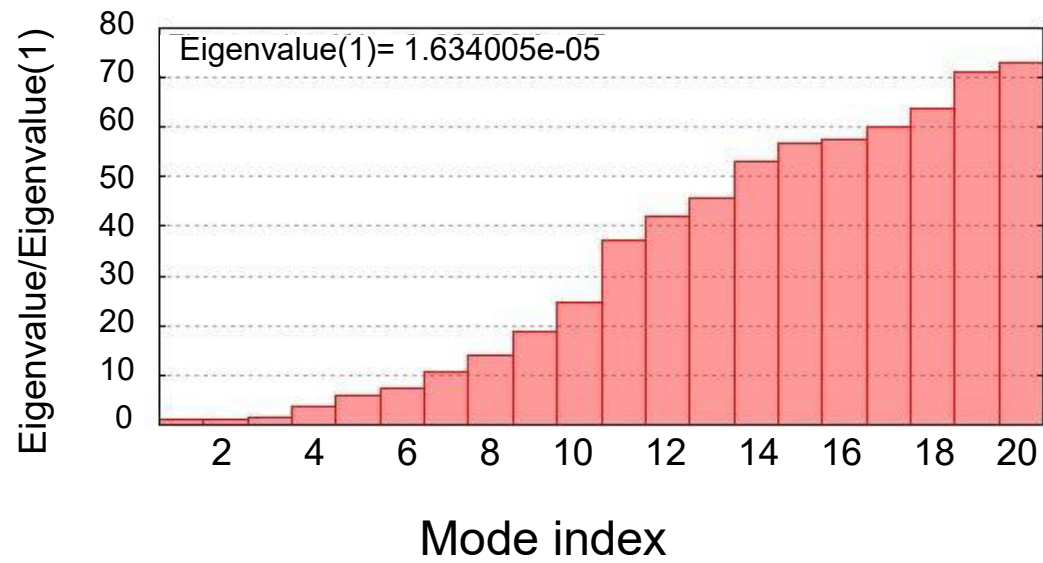

c

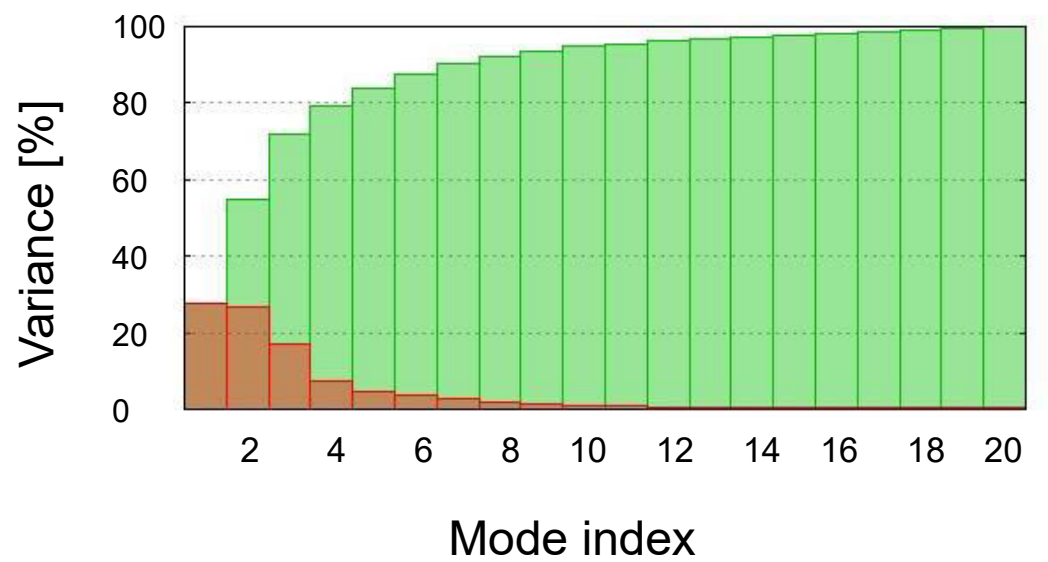

d

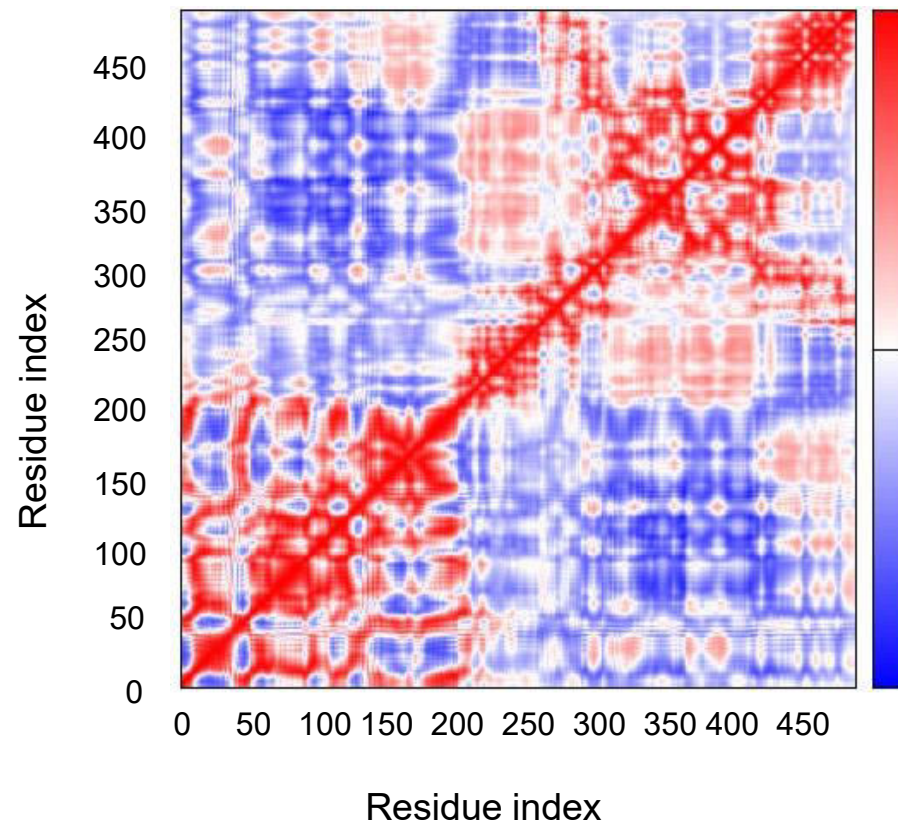

e

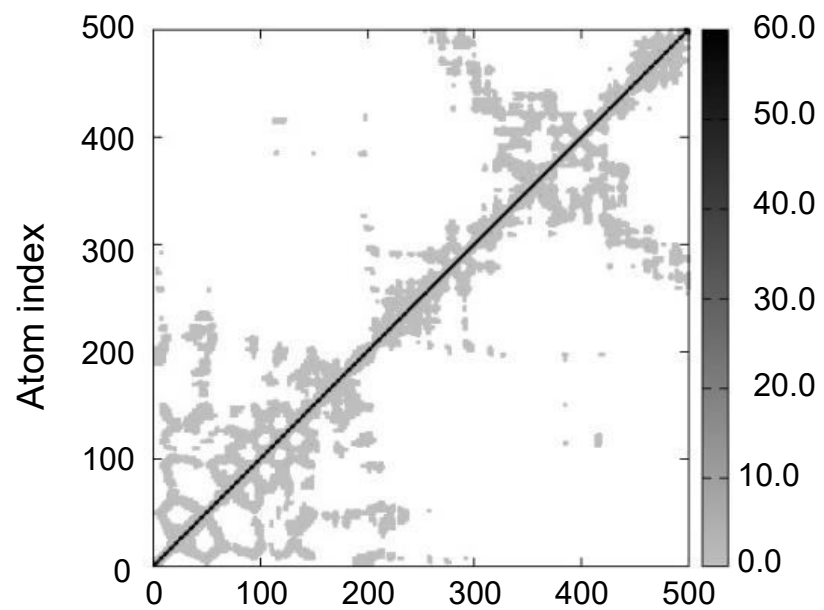

f

Supplement: Supplementary Materials — Table S1 provides information about which amino acid substitution may alter the molecular mechanism of the ADAM33 enzyme. Tables S2-S4 provide information on possible posttranslational modifications of amino acid substitution within the ADAM33 enzyme. Finally, Figures S1-S7 portray the normal mode analysis of wild-type and mutated proteins including B-factor, deformability, eigenvalue, variance, and elastic network. [file 1089722.f1.zip › Figure S3.pdf]

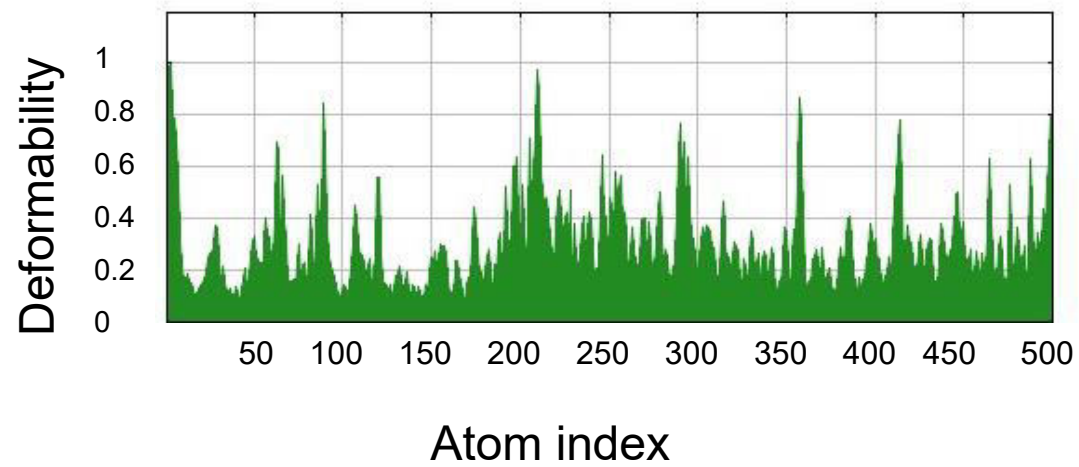

a

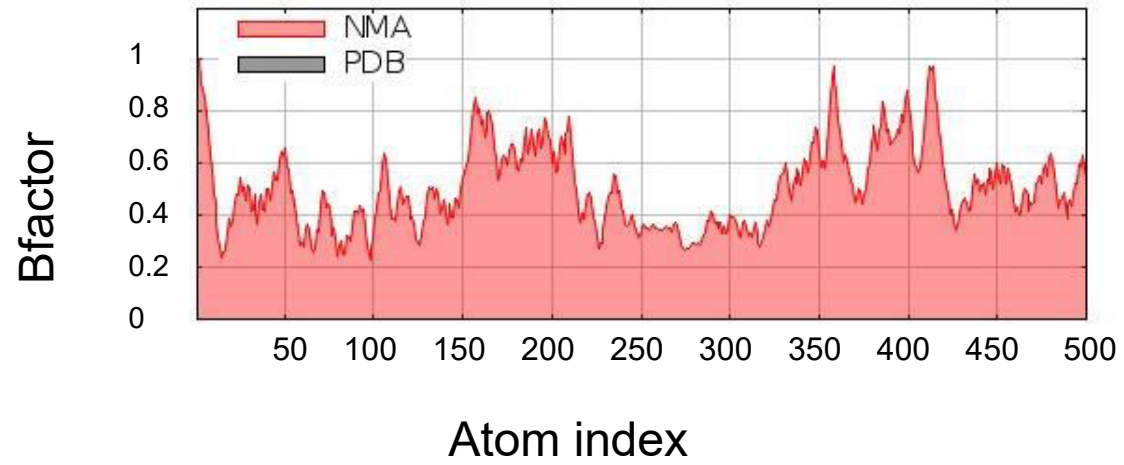

b

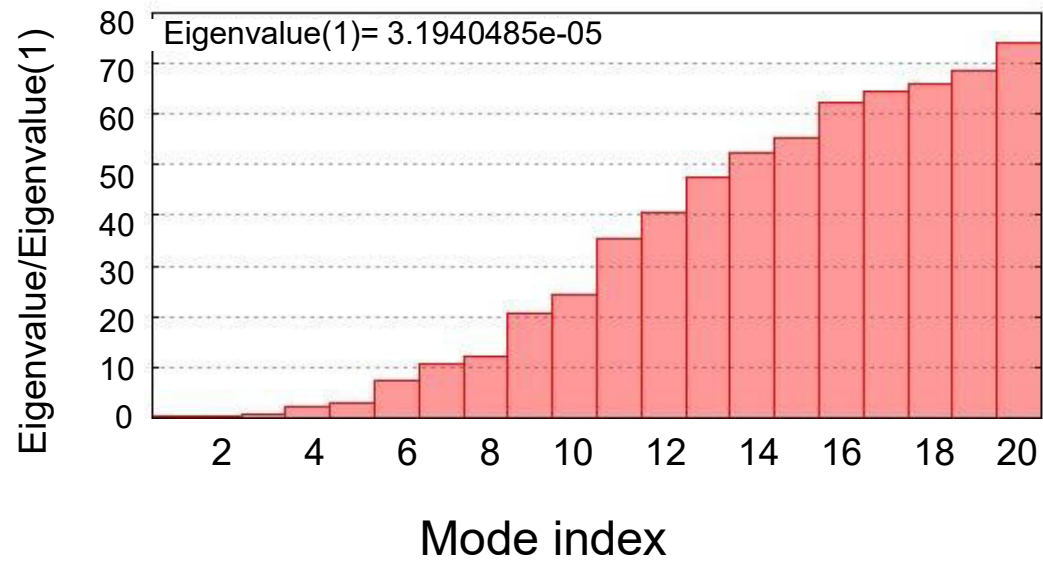

c

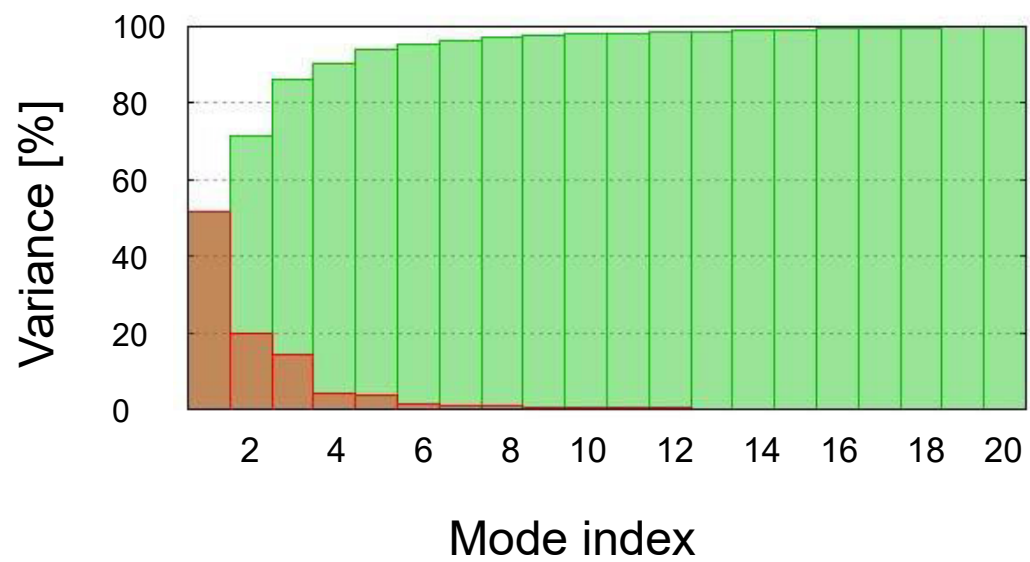

d

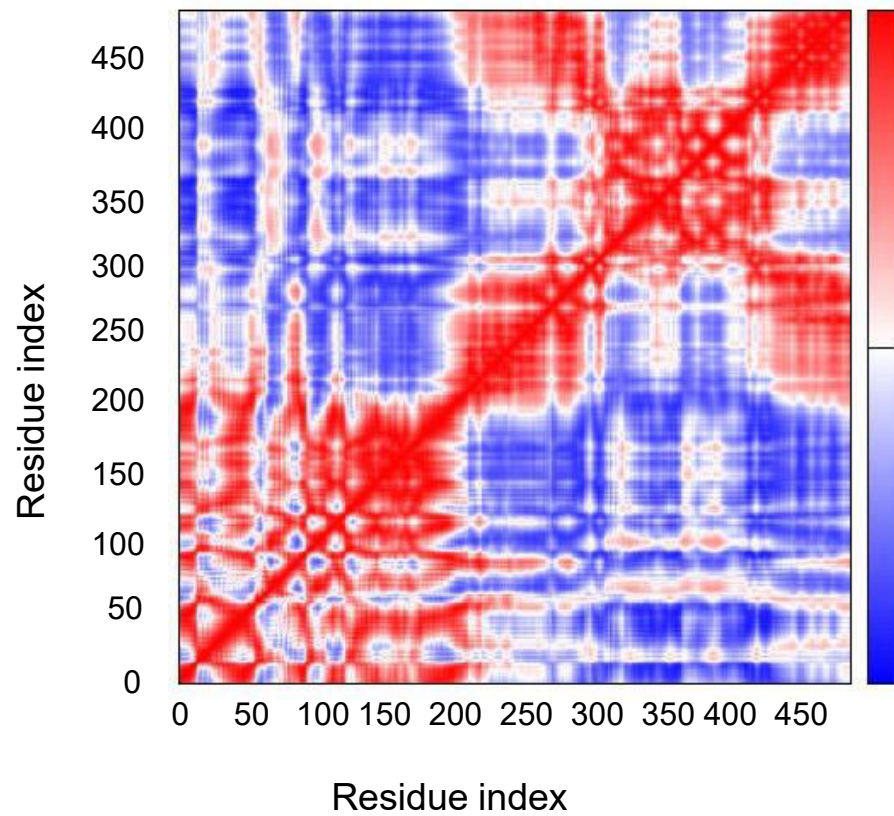

e

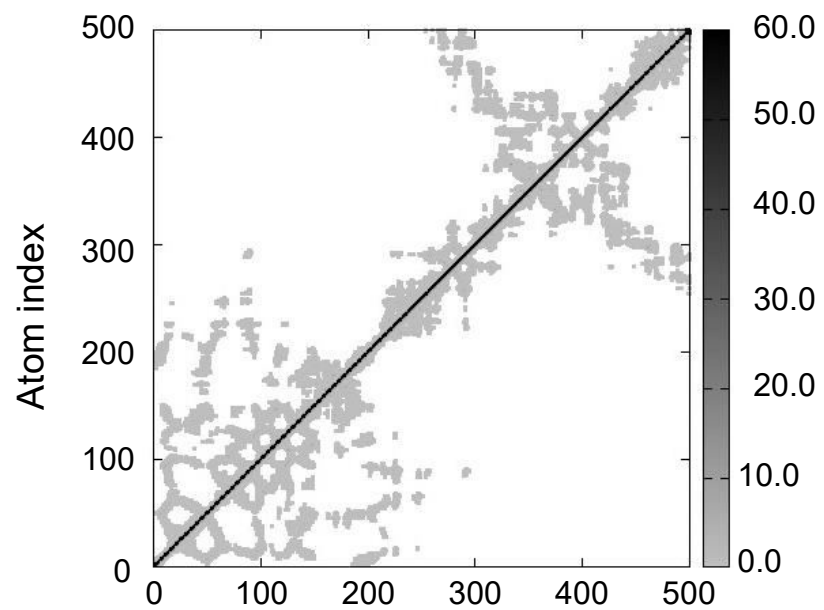

f

Atom index

Supplement: Supplementary Materials — Table S1 provides information about which amino acid substitution may alter the molecular mechanism of the ADAM33 enzyme. Tables S2-S4 provide information on possible posttranslational modifications of amino acid substitution within the ADAM33 enzyme. Finally, Figures S1-S7 portray the normal mode analysis of wild-type and mutated proteins including B-factor, deformability, eigenvalue, variance, and elastic network. [file 1089722.f1.zip › Figure S4.pdf]

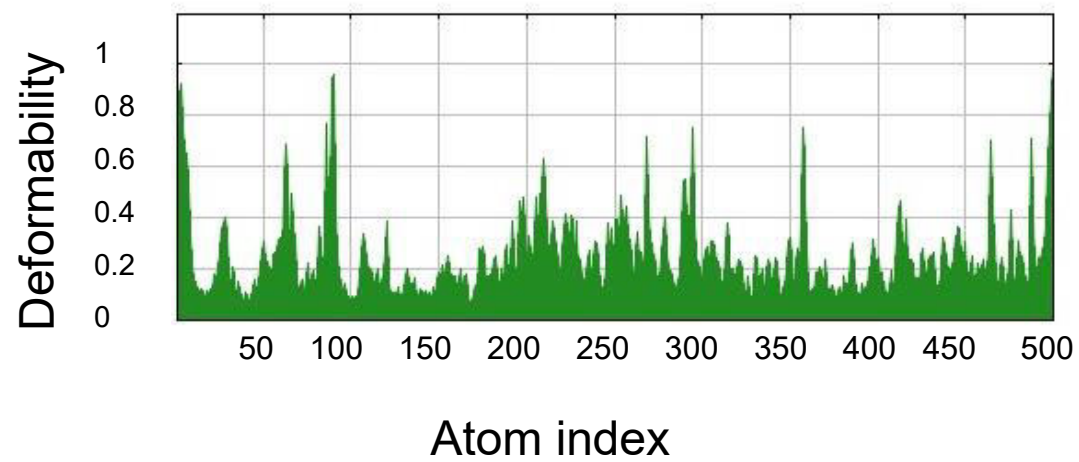

a

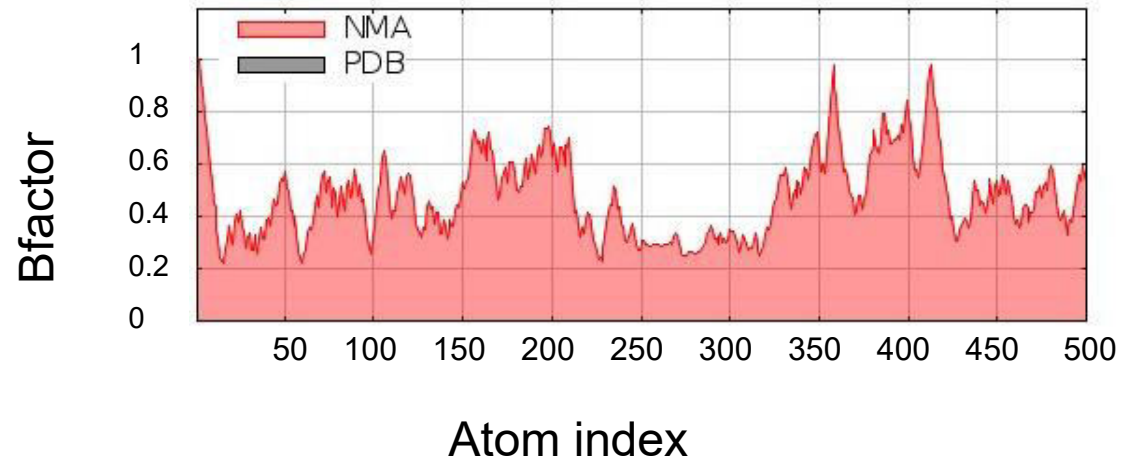

b

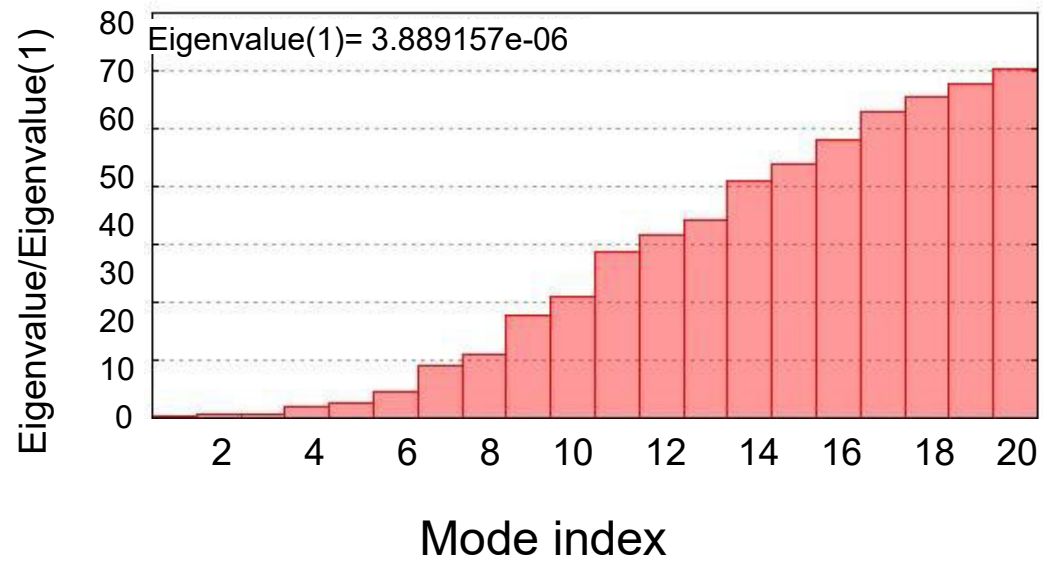

c

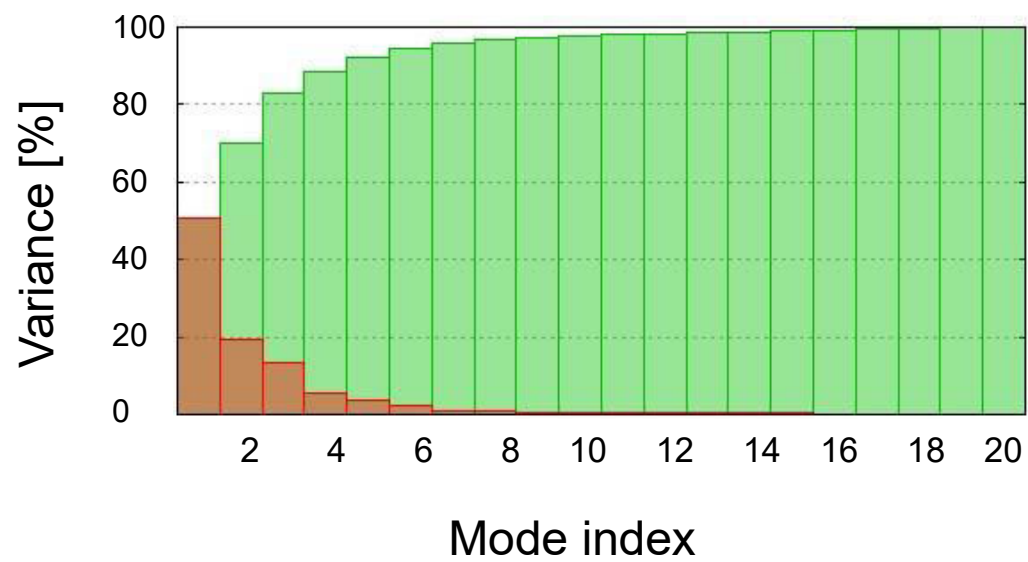

d

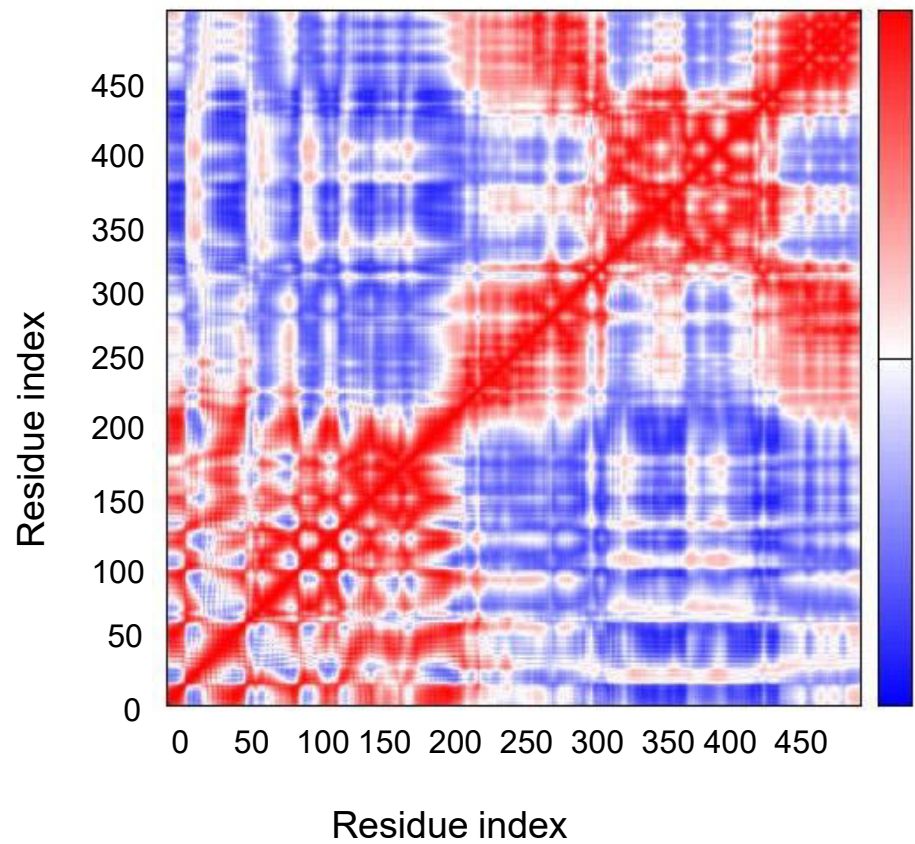

e

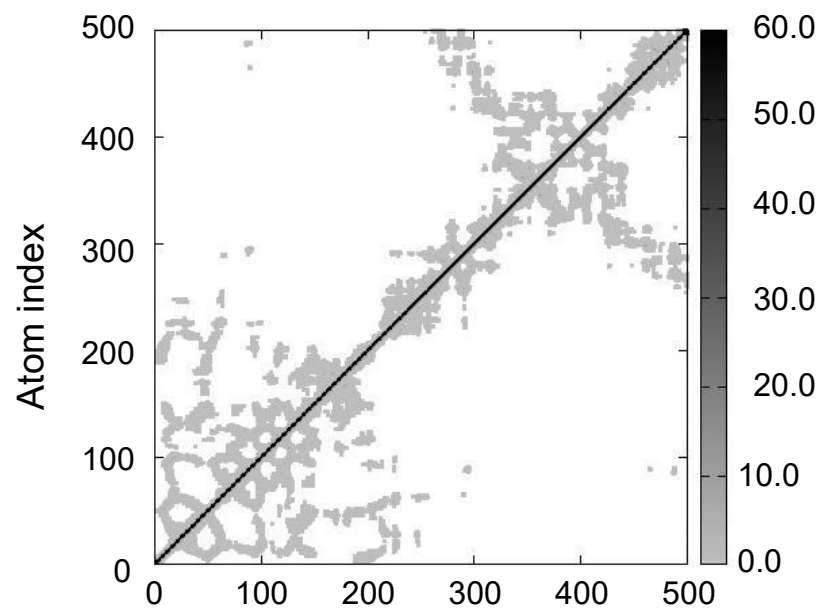

f

Atom index

Supplement: Supplementary Materials — Table S1 provides information about which amino acid substitution may alter the molecular mechanism of the ADAM33 enzyme. Tables S2-S4 provide information on possible posttranslational modifications of amino acid substitution within the ADAM33 enzyme. Finally, Figures S1-S7 portray the normal mode analysis of wild-type and mutated proteins including B-factor, deformability, eigenvalue, variance, and elastic network. [file 1089722.f1.zip › Figure S5.pdf]

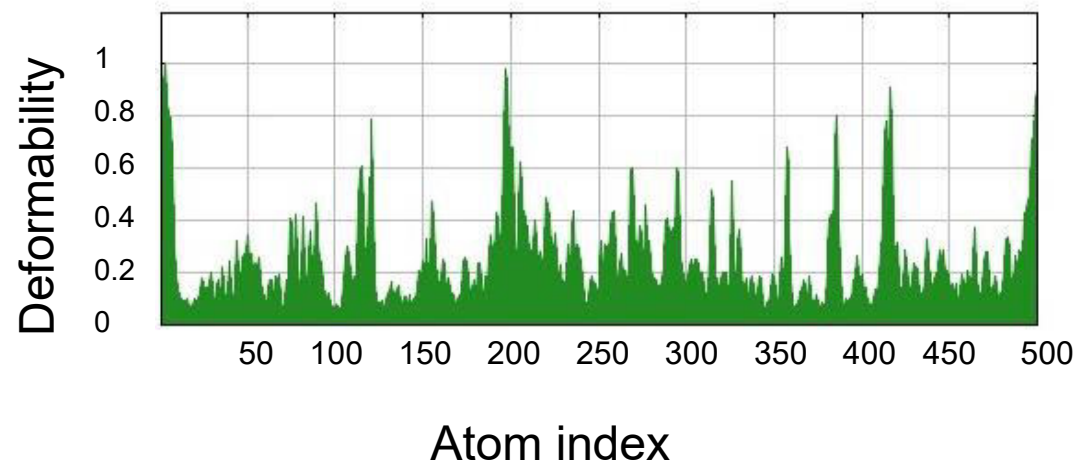

a

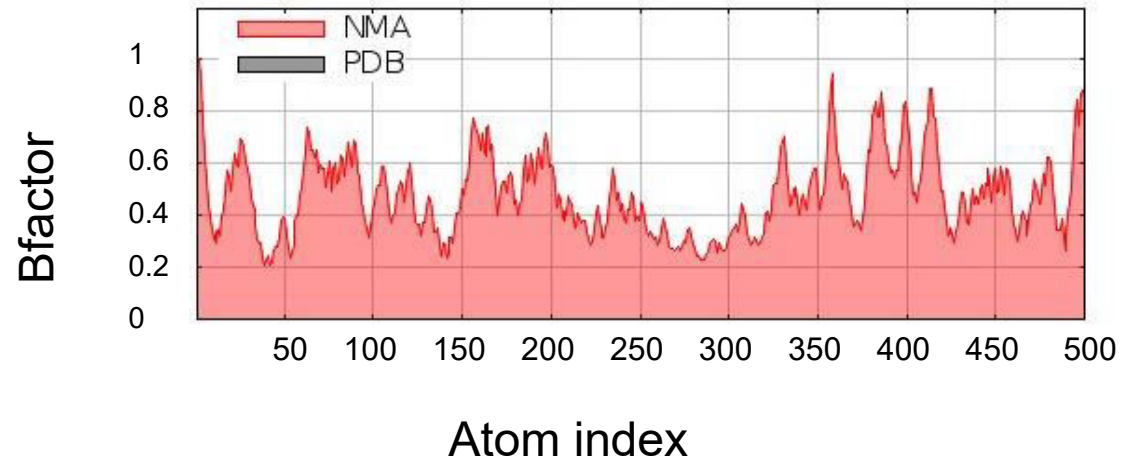

b

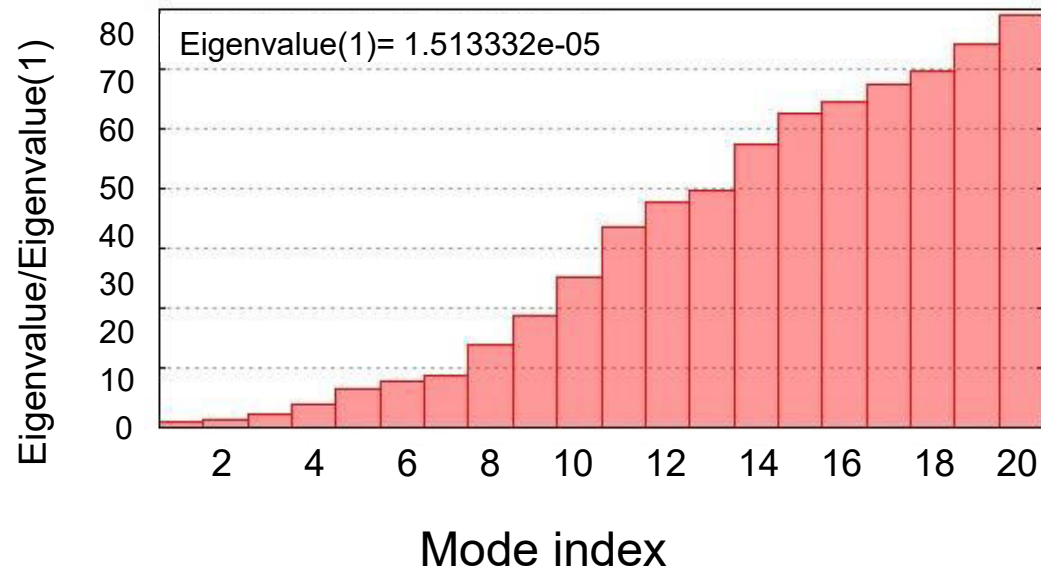

c

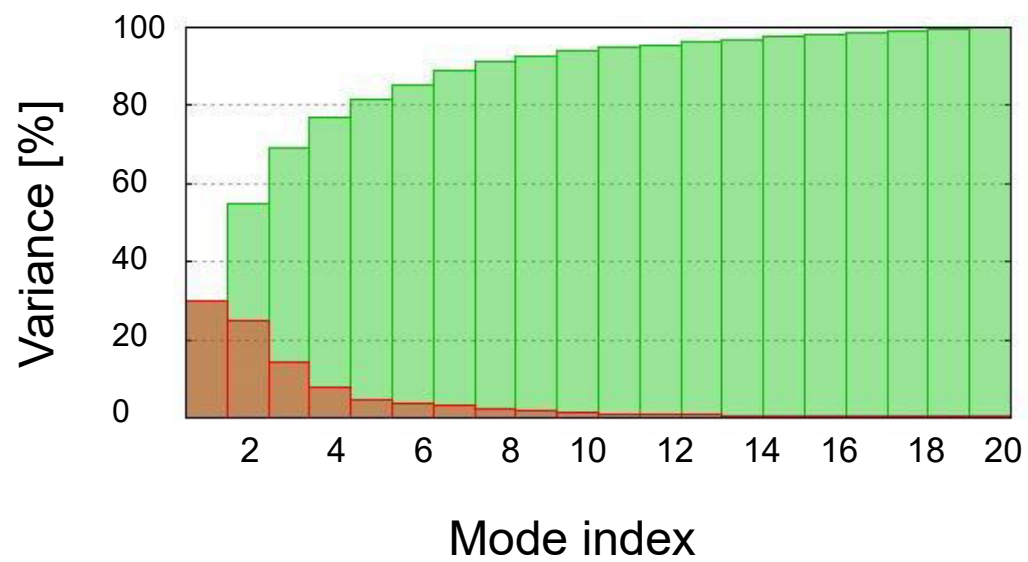

d

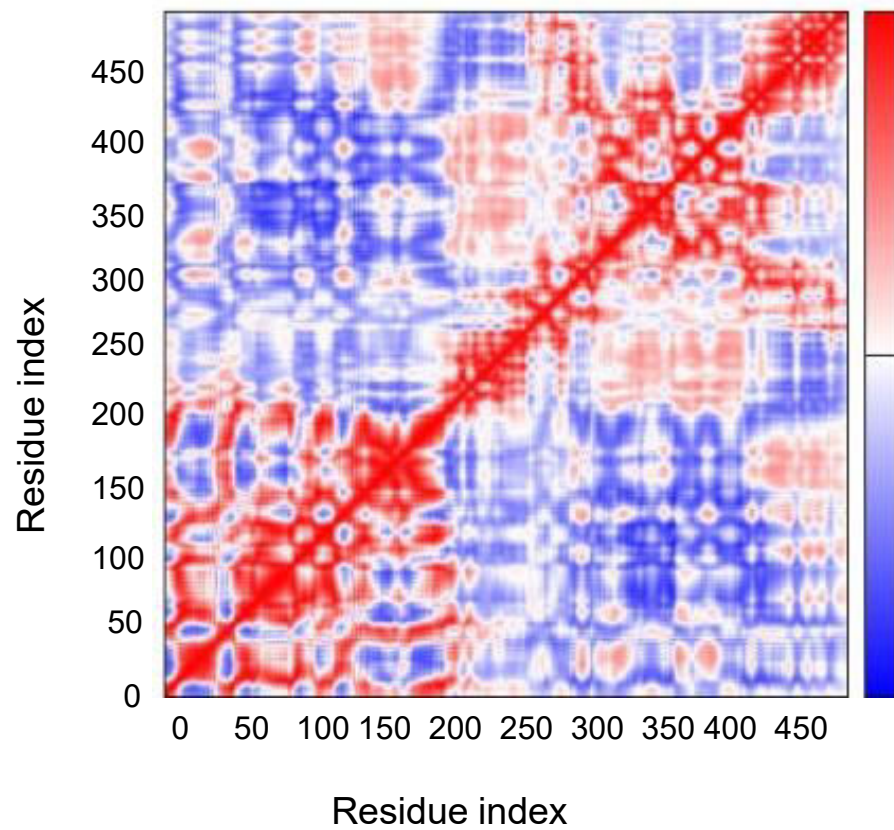

e

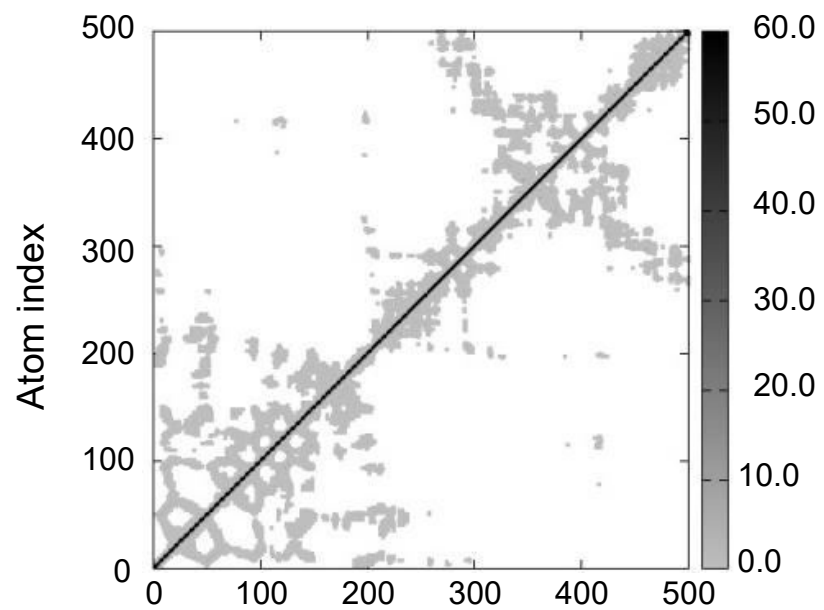

f

Atom index

Supplement: Supplementary Materials — Table S1 provides information about which amino acid substitution may alter the molecular mechanism of the ADAM33 enzyme. Tables S2-S4 provide information on possible posttranslational modifications of amino acid substitution within the ADAM33 enzyme. Finally, Figures S1-S7 portray the normal mode analysis of wild-type and mutated proteins including B-factor, deformability, eigenvalue, variance, and elastic network. [file 1089722.f1.zip › Figure S6.pdf]

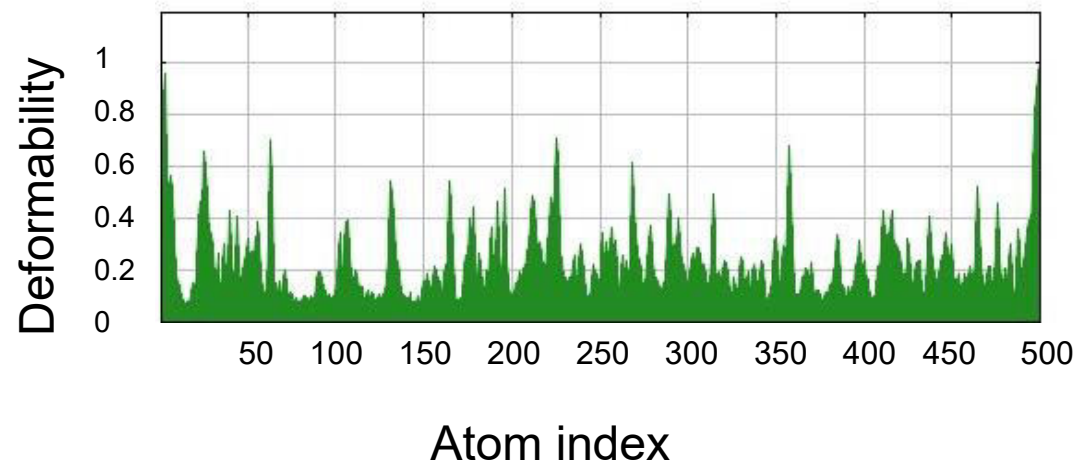

a

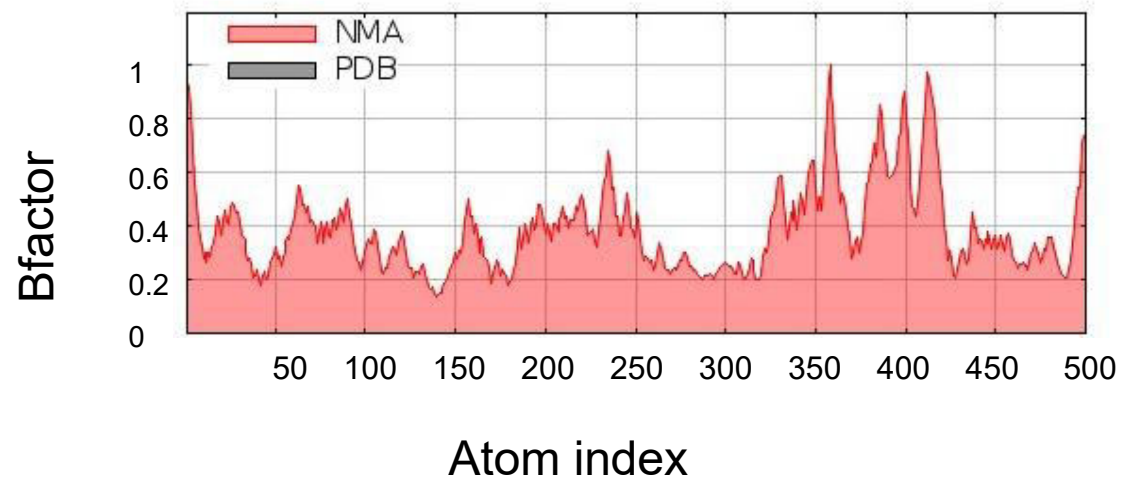

b

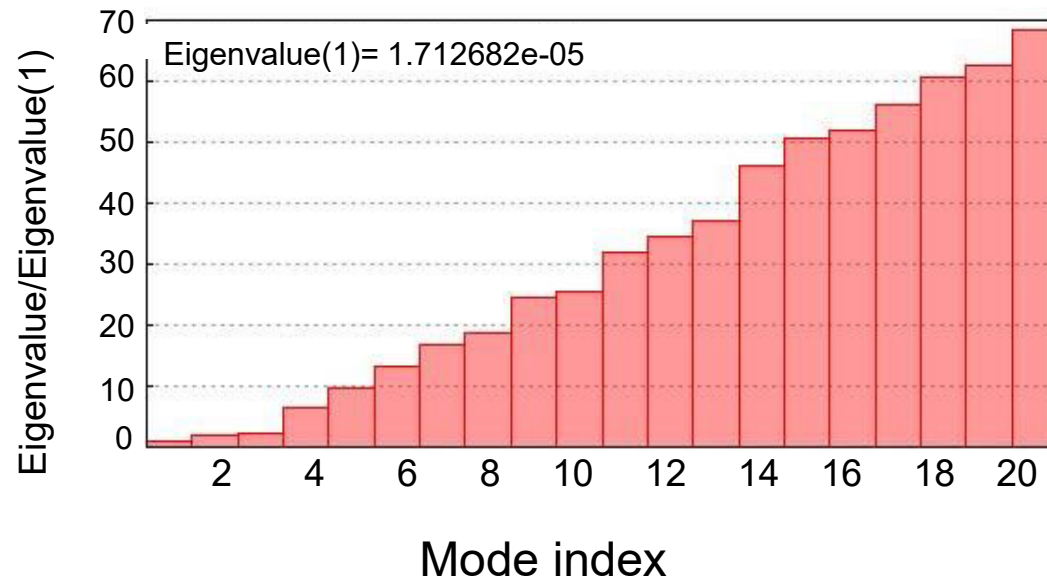

c

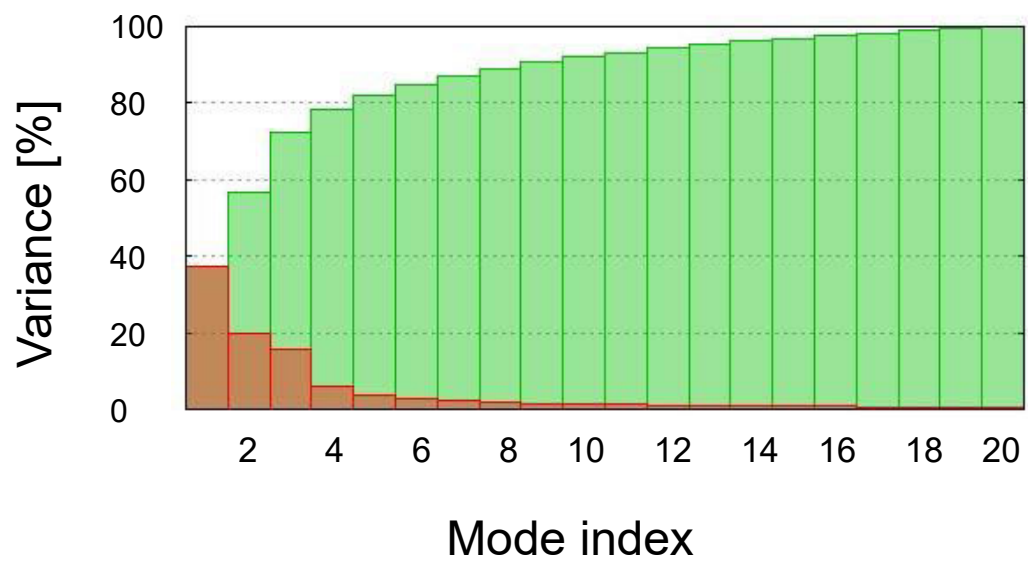

d

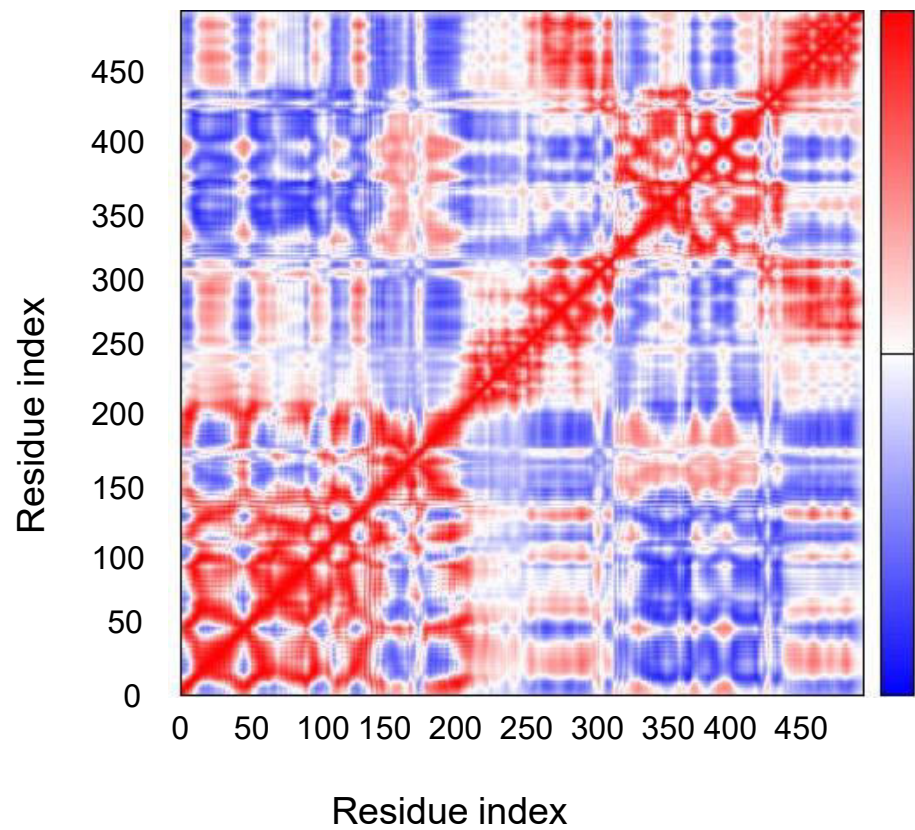

e

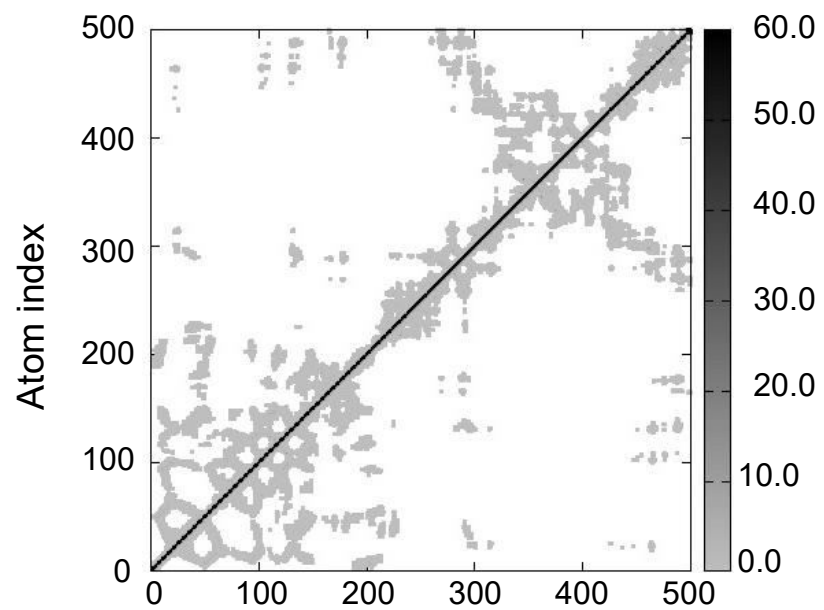

f

Atom index

Supplement: Supplementary Materials — Table S1 provides information about which amino acid substitution may alter the molecular mechanism of the ADAM33 enzyme. Tables S2-S4 provide information on possible posttranslational modifications of amino acid substitution within the ADAM33 enzyme. Finally, Figures S1-S7 portray the normal mode analysis of wild-type and mutated proteins including B-factor, deformability, eigenvalue, variance, and elastic network. [file 1089722.f1.zip › Figure S7.pdf]
